# microRNAs and Their Roles in Plant Development

**DOI:** 10.3389/fpls.2022.824240

**Published:** 2022-02-18

**Authors:** Qingkun Dong, Binbin Hu, Cui Zhang

**Affiliations:** ^1^Key Laboratory of Plant Molecular Physiology, CAS Center for Excellence in Molecular Plant Sciences, Institute of Botany, Chinese Academy of Sciences, Beijing, China; ^2^College of Life Sciences, University of Chinese Academy of Sciences, Beijing, China

**Keywords:** microRNA, plant development, microRNA movement, hormone, crop yield

## Abstract

Small RNAs are short non-coding RNAs with a length ranging between 20 and 24 nucleotides. Of these, microRNAs (miRNAs) play a distinct role in plant development. miRNAs control target gene expression at the post-transcriptional level, either through direct cleavage or inhibition of translation. miRNAs participate in nearly all the developmental processes in plants, such as juvenile-to-adult transition, shoot apical meristem development, leaf morphogenesis, floral organ formation, and flowering time determination. This review summarizes the research progress in miRNA-mediated gene regulation and its role in plant development, to provide the basis for further in-depth exploration regarding the function of miRNAs and the elucidation of the molecular mechanism underlying the interaction of miRNAs and other pathways.

## Introduction

RNA is one of the four major macromolecules of life and is essential in the regulation and expression of genes. RNA can be divided into two groups: coding and non-coding RNAs. In plants, 24 nt small interfering RNAs (siRNA) and 21 nt microRNAs (miRNA) have the highest expression abundance of small non-coding RNAs. siRNAs were first discovered in plants and are involved in the transcriptional gene silencing and post-transcriptional gene silencing (PTGS) pathway in plants (Hamilton and Baulcombe, 1999; Sijen et al., 2001; Pal-Bhadra et al., 2002) and RNA interfering pathway in animals (Elbashir et al., 2001).

miRNAs were first identified from nematodes (*Caenorhabditis elegans*) by Victor Ambros lab in collaboration with Gary Ruvkun lab, who confirmed that a miRNA (Lin-4) has a role in regulating the temporal developmental of nematode larvae (Lee et al., 1993; Wightman et al., 1993; Fire et al., 1998; Stricklin et al., 2005). Since then, miRNAs have been reported in *Drosophila*, nematodes, mammals, and plants. In plants, 22 nt miRNA is able to cut the target mRNA and the cleavage product can be further processed by RNA-DEPENDENT RNA POLYMERASE 6 (RDR6) and DICER-LIKE 4 to produce secondary 21 nt siRNA. In addition, the symmetric miRNA/miRNA* can be processed by DCL2 and generate secondary 22 nt miRNAs. These siRNAs are called phased siRNAs (PhasiRNAs) because they are the endogenous plant siRNAs with phase arrangement structure characteristics (Borges and Martienssen, 2015). PhasiRNA can be divided into cis-acting siRNA and trans-acting siRNA (ta-siRNA; Chen et al., 2010; Zhai et al., 2011; Arikit et al., 2014; Deng et al., 2018).

miRNAs are demonstrated to be vital in plant development. They are usually transcribed by RNA Polymerase II (Pol II) into pri-miRNAs. These pri-miRNAs are cleaved by a class of RNase-III nucleases called Dicer-like proteins, after which they combine with ARGONAUTE (AGO) family proteins to form the RNA-induced silencing complexes (RISCs). RISCs are then involved in the expression and regulation of target genes (Song et al., 2019). miRNAs act in the regulation of meristem characteristics, leaf polarity, flowering patterns. etc. Mutations in miRNA transcription or processing complexes usually have multiple effects on plant from and function, indicating that miRNAs are important to coordinate plant development. For example, the roles of HD-ZIP III-miR165/166 pathway are important in the development of vascular, meristem, and leaf polarity, and the roles of miR156/miR172 are important in flowering time and flower pattern (D’Ario et al., 2017; Ramachandran et al., 2017; Du et al., 2020; Ma et al., 2020; Lian et al., 2021; Yadav et al., 2021). During plant development, endogenous miRNAs play an important role in gene regulation by targeting related target genes. Several miRNAs function through interactions with hormones. Many components in hormone signaling are targets of miRNAs, and the interactions of these components and the miRNAs enable plants to regulate their growth, development, and differentiation rapidly and effectively. This signaling is done by selecting miRNAs as intermediates to control hormone responses or, conversely, by using hormones to regulate specific miRNA levels (Jodder, 2020; Li et al., 2020; Yu and Wang, 2020). There is evidence indicating that miRNAs can diffuse in tissues as inhibitor signals, so they play an elaborate role in tissue differentiation (Chen and Rechavi, 2021). Here we will summarize the role of miRNAs in the aspects of biogenesis, action mechanism, function in specific tissues, interaction with hormones, and movement to understand how they regulate plant development. miRNAs and their targets involved in plant development are listed in Table 1.

**Table 1:** miRNAs, the targets, and their roles in plant development.

miRNA	Target	Target function	Species	References
miR156	SPL family	Plastochron length, promoting flowering; Leaf development, root development, secondary metabolism and abiotic stress; tillering and corn development in *Zea mays*	*Arabidopsis* and *Zea mays*	Aukerman and Sakai, 2003; Chuck et al., 2007a, 2010; Wang et al., 2008; Xu et al., 2016b; Dai et al., 2018
miR159	GAMYB or GAMYB-like gene	Male reproductive development, seed development, vegetative tissues and reproductive development	*Arabidopsis*	Allen et al., 2007; Millar et al., 2019
miR160	ARFs	Embryo, leaf and root development, hypocotyl elongation	*Arabidopsis*, Medicago truncatula and *Zea mays*	Bustos-Sanmamed et al., 2013; Lopez-Ruiz et al., 2019; Yang et al., 2019; Dai et al., 2021
miR164	NAC family	Meristem boundary identity, Auxiliary meristem formation, leaf and flower development, lateral root initiation	*Arabidopsis*, *Zea mays* and *Oryza*	Li et al., 2003; Laufs et al., 2004; Hibara et al., 2006; Raman et al., 2008; Zheng et al., 2019; Wang et al., 2021b
miR165/166	HD-ZIP III	Maintaining meristematic cells, adaxial identity of leaves, lateral root growth, and procambium identity	*Arabidopsis*	Williams et al., 2005; Jia et al., 2015; Merelo et al., 2016; Yan et al., 2016
miR167	ARFs	Development of male organ、 roots、 stems、 leaves and flowers, flowering time, embryonic development, seed development and stress response, defense against pathogens	*Arabidopsis* and *Oryza*	Wu et al., 2006; Liu et al., 2012; Yao et al., 2019; Caruana et al., 2020
miR169	CBF and NF-YA family	Enhancer of C homeotic gene transcription and root architecture	*Arabidopsis*, *Antirrhinum majus* and *Zea mays*	Cartolano et al., 2007; Sorin et al., 2014; Xu et al., 2014; Xing et al., 2021
miR171	SCL	Chlorophyll biosynthesis, phase transitions and floral meristem determinacy	*Arabidopsis*, barley	Curaba et al., 2013; Ma et al., 2014; Li et al., 2021
miR172	AP2 family	Represses flowering, flower meristem identity and patterning; vegetative phase change, carpel and stamen development; flower opening, tuberization and *salt tolerance*	*Arabidopsis*, *Z. mays*, *Oryza,H. vulgare*, and *S. tuberosum*	Chuck et al., 2007b; Martin et al., 2009; Wu et al., 2009; Nair et al., 2010; Wollmann et al., 2010; Zhu and Helliwell, 2011; Cheng et al., 2021a; Lian et al., 2021; Werner et al., 2021
miR319	TCP family	Leaf development and senescence, organ curvature, and hormone biosynthesis and signaling.	*Arabidopsis* and *Solanum lycopersicum*	Ori et al., 2007; Schommer et al., 2014; Koyama et al., 2017; Bresso et al., 2018
miR390	TAS3	ta-siRNA biogenesis for ARF repression and indirect miR165/166 regulation, lateral root growth, leaf patterning	*Arabidopsis*	Fahlgren et al., 2006; Marin et al., 2010; Endo et al., 2013; Dastidar et al., 2019
miR393	TIR1 and AFB	Auxin homeostasis, lateral root growth, leaf shape/number	*Arabidopsis* and *Oryza*	Parry et al., 2009; Chen et al., 2011; Windels and Vazquez, 2011; Lu et al., 2018; Wang et al., 2018
miR394	LCR	Meristematic identity suppression *via* WUS downregulation, leaf inclination and architecture,	*Arabidopsis*	Baumann, 2013; Knauer et al., 2013; Qu et al., 2019
miR396	GRF	Cell proliferation in leaves, disease-resistance, somatic embryogenesis, grain size and panicle branching	*Arabidopsis*, *Medicago*, and *Oryza*	Debernardi et al., 2012; Bazin et al., 2013; Liu et al., 2014a; Chandran et al., 2018; Szczygiel-Sommer and Gaj, 2019; Liebsch and Palatnik, 2020; Zhang et al., 2020
mir397	OsLAC	Grain yield, panicle branches	*Oryza*	Zhang et al., 2013
miR824	AGL16	Stomatal patterning	*Arabidopsis*	Bergmann and Sack, 2007
miR828 and miR858	MYBs	Fiber development, anthocyanin, and flavonol accumulation	Cotton, grapes	Guan et al., 2014; Tirumalai et al., 2019
miR847	IAA28	Lateral root formation	*Arabidopsis*	Wang and Guo, 2015
miR857	LACCASE7	Secondary growth	*Arabidopsis*	Abdel-Ghany and Pilon, 2008; Zhao et al., 2015
TAS3	ARF3/4 and (only in mosses) AP2-like	Vasculature development, Leaf polarity / phase transition	All land plants	Fahlgren et al., 2006; Jing et al., 2017

## Biosynthesis and Action Mechanism of miRNAs in Plants

Most of miRNAs are a kind of conserved endogenous small RNA, which plays an important regulatory role after eukaryotic gene transcription (Rodriguez et al., 2010). Most metazoan miRNA genes exist in thousands of introns or exons, whereas plant miRNA genes exist between genes. In addition to this, the secondary structures and lengths of miRNA are greatly different among plant species (Voinnet, 2009). Animal miRNAs exist in clusters along the genome, and they can be co-transcribed in the form of poly-cistrons (Ha and Kim, 2014). In contrast, plant miRNA genes are rarely arranged in series (Kim, 2005; Zhang et al., 2008). Like protein-coding genes, miRNAs start by being transcribed in the nucleus by Pol II to form pri-miRNAs, which range in length from several hundred to several thousand nucleotides and have a 5′ cap and a 3′ poly-A tail (Jones-Rhoades and Bartel, 2004; Lee et al., 2004; Jones-Rhoades et al., 2006). Under the action of DCL1, pri-miRNAs are cleaved into pre-miRNAs, which are ~70 nt – 350 nt. These pre-miRNAs are further formed by the interaction of the DCL1 enzyme, RNA-binding protein HYL1 (Hyponastic Leaves 1), and C2H2 zinc finger protein SE (Serrate) on pre-miRNA (Kurihara and Watanabe, 2004; Jones-Rhoades et al., 2006) into mature miRNAs. The mature miRNAs have 2 bases protruding at the 3′ end (miRNA double-stranded complex). This miRNA complex is methylated at the 2′-OH position of its 3′ end under the action of HUAENHANCER1 protein to prevent degradation (Li et al., 2005). Most of the methylated miRNA complexes are transported into the cytoplasm with the help of plant homolog of exportin-5, HASTY (HST; Park et al., 2005; Brioudes et al., 2021). The RNA-induced RISC, generated by miRNA, is eventually produced in the cytoplasm (Park et al., 2005; Jones-Rhoades et al., 2006). Recent studies showed that RISC can be assembled in the nucleus and exported to the cytosol by EXPO1 (Bologna et al., 2018), and HST also regulates pri-miRNA transcription and processing (Cambiagno et al., 2021). In the RISC complex, the AGO protein is the most important structural protein. It contains four domains: the N-terminal domain (N), the PIWI/Argonaute/Zwille (PAZ) domain, the MID domain, and the P-element-induced wimpy tested (PIWI) domain. The PAZ domain can bind to RNA and the PIWI domain with RNase H activity. 10 different types of AGO proteins have been found in *Arabidopsis thaliana*; most of them contain catalytic reaction residues. Of these different AGO proteins, ARGONAUTE1 (AGO1; Baumberger and Baulcombe, 2005; Qi et al., 2005), AGO2 (Carbonell et al., 2012), AGO4 (Qi et al., 2006), AGO7 (Montgomery et al., 2008), and AGO10 (Ji et al., 2011; Zhu et al., 2011) have been demonstrated in the gene silencing pathway of target RNAs. AGO1 protein is involved in PTGS as the main component of RISC that binds to a short guide RNA such as miRNA or siRNA. AGO4 and AGO6 are mainly involved in the repeat-associated siRNA pathway, and AGO7 plays a role in the formation of ta-siRNA (Vaucheret, 2008; Duan et al., 2015; Singh et al., 2015; Fang and Qi, 2016).

Studies have shown that mature miRNAs inhibit the translation of target genes, regulate the expression of plant genes by complementary pairing with coding region, some binding to 3′UTR and 5′UTR of the target mRNA, or regulate the expression of genes by cutting target gene mRNA at the post-transcriptional level. This inhibition by mature miRNAs alters the morphogenesis of plant organs, growth, development, hormone secretion, signal transduction, and the ability of plants to respond to external stress and environmental factors (Liu et al., 2009a; Yokotani et al., 2009; Naqvi et al., 2012). miRNA in plants is highly complementary to its target mRNA, so its main mode of action is cleavage. The translation inhibition pathways in plants have only been found in recent years. The cleavage and inhibition mechanisms are mostly coordinated depending on the complementarity between miRNAs and their targets (Brodersen et al., 2008; Yu et al., 2017; O’Brien et al., 2018).

## The Function of miRNAs in Plant Growth and Development

The regulation of plant growth and development is very precise and is influenced by both internal genetic information and the external environmental factors. Normal expression of miRNAs is necessary for the growth and development of plants. Previous studies have shown that miRNA widely regulates plant growth and development.

### The Role of miRNAs in the Shoot Meristem

Unlike animals, plants can continuously produce new organs throughout their life cycle. Their apical meristem forms in embryo and has a group of stem cells with multidirectional differentiation potential and the ability to self-replicate. During the development of a plant, the shoot apical meristem (SAM) plays a central role in the formation and development of its aboveground organs. The STM (shoot meristemless)-WUS (Wuschel)-CLV (Clavata) pathway plays a key role in the maintenance of meristem activity (Schoof et al., 2000; Gaillochet and Lohmann, 2015; Somssich et al., 2016). To some extent, the same mechanisms are also demonstrated in flower meristems.

**Figure 1:**
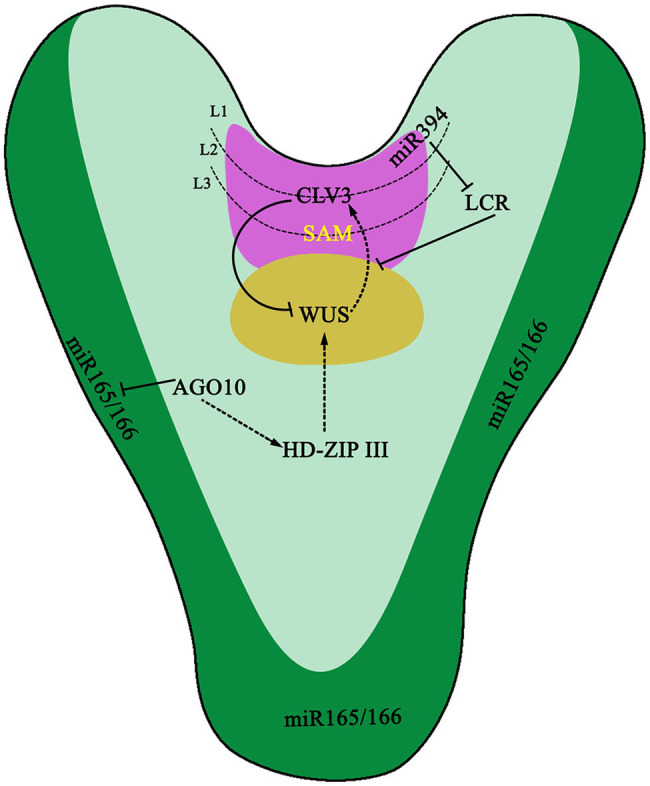
The function of miRNAs in embryo. miR394 expresses in the L1 layer of shoot apical meristem (SAM) and then moves to L3 layer to target *Leaf Curling Responsiveness* (*LCR*) gene. LCR further regulates CLAVATA-WUSCHEL (CLV-WUS) negative feedback loop for proper SAM development and specification. ARGONAUTE10 (AGO10) specifically sequesters miR166/165 to upregulate Class III homeodomain leucine zipper transcription factors (HD-ZIP III TFs) to maintain SAM development. The dotted arrows represent a proposed positive regulation, whereas lines with perpendicular end bars indicate negative regulation.

miRNA plays a central role in the regulation of gene expression networks, orchestrating the establishment and the maintenance of the SAM by targeting and regulating multiple genes in the STM-WUS-CLV signaling pathway (Figure 1). miR394 is generated in the L1 layer on the surface of the SAM and diffuses down to the Organizing Center (OC; Figure 1). In the OC, expression of Leaf Curling Responsiveness (*LCR*) is inhibited (Knauer et al., 2013) and directly results in the downregulation of *WUS*, a SAM-specific gene (Song et al., 2012). Although the concentration of miR394 in the L1 layer is higher than that in the OC layer, the inhibitory effect of miR394 on LCR only occurs in OC, implying that an exact concentration of miR394 is of great importance to its function in *A. thaliana* (Knauer et al., 2013). Meanwhile, there are diversified functions for stem cell regulation mediated by miR394-LCR (Kumar et al., 2019). AGO10 can specifically bind to miR165/166 and ultimately promote the expression of *HD-ZIP III*. *HD-ZIP III* is an important transcription factor family that regulates SAM in *A. thaliana* and is a target of miR165/166. When miR166/165 does not bind to AGO10 or the *AGO10* gene is knocked out, the meristematic tissue of plants is destroyed. AGO1 antagonizes AGO10 in the binding of miR166/165. When miR166/165 binds to AGO1, plants will decrease the expression of the *HD-ZIP III* genes and terminate SAM development. Recent studies indicate that the interaction between AGO10 and miR165/166 depends solely on the structure of miR165/166 and is independent of the catalytic activity of AGO10 (Zhu et al., 2011).

### The Role of miRNAs in Leaf Development

**Figure 2:**
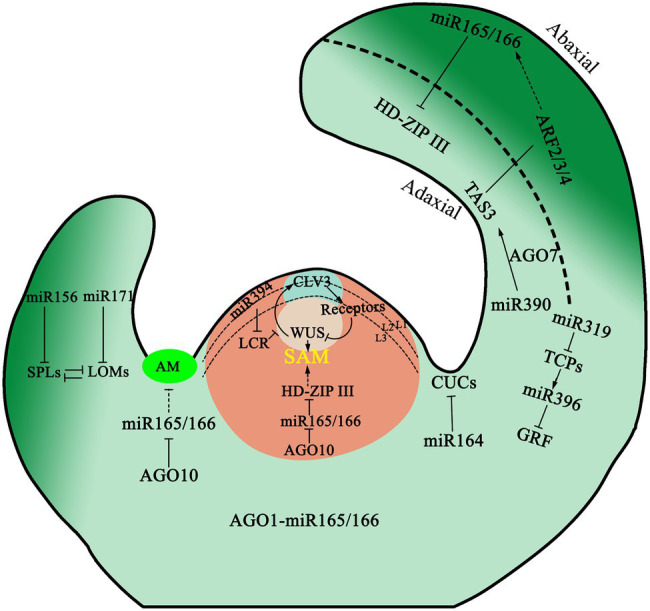
Model for the role of miRNAs in shoot apex. miR394 synthesized at the protoderm represses *LCR* in subtending cells, which leads to the activation of the WUSCHEL (WUS) transcription factor to maintain stem cell identity and *CLAVATA3 (CLV3) peptide* expression. ARGONAUTE10 (AGO10) specifically sequesters miR165/166 and antagonizes its activity in the meristematic cells, thus regulating SAM and AM development. ARGONAUTE1 (AGO1) is expressed ubiquitously in the apex, recruit miR165/166 to form RNA-induced silencing complex (RISC). The adaxial and abaxial domains of leaves are established during leaf primordia emergence. HD-ZIP III transcription factors are restricted to the adaxial side by the action of miR165/166. In turn, AUXIN RESPONSE FACTOR 2/3/4 are restricted to the abaxial side by the action of TAS3 ta-siRNA. Two NAC-domain transcription factors are post-transcriptionally regulated by miR164 in embryonic meristem initiation, boundary size control, and cotyledon establishment. miR319 and miR396 target several *TEOSINTE BRANCHED1/CYCLOIDEA/PROLIFERATING CELL FACTOR* (*TCP*) and *Growth-Regulating Factor* (*GRF*) genes, respectively, and act coordinately to control leaf cell proliferation and differentiation. miR156 and miR171 synergistically regulate trichome initiation by targeting *SQUAMOSA PROMOTER BINDING PROTEIN-LIKE* (*SPL*) and *LOST MERISTEMS* (*LOM*), respectively. Arrows indicate positive regulation, whereas the dotted lines with perpendicular end bars represents a hypothesized negative regulation.

Leaf development includes the differentiation of leaf primordium from the SAM and the subsequent development of leaf blades. In these processes, various regulatory factors are involved. Organogenesis in the SAM is determined by the distribution and polar transport of auxin (Veit, 2009). The target genes of miR160, namely *ARF10*, *ARF16*, and *ARF17* in the auxin response factors (ARF) family affect leaf development by regulating auxin response. Mutants *arf10* and *arf17* of *A. thaliana* which are resistant to miR160 cleavage, have an abnormal number of cotyledons, and the edge of the leaves was serrated and curled upward (Liu et al., 2007). At the same time, leaf genesis is regulated by several transcription factors, such as the expression of *MYB DOMAIN PROTEIN* (*MYB*) transcription factor, in leaf primordium. These specific ASYMMETRIC LEAVES1/ROUGH SHEATH2/PHANTASTICA gene families can be used as a transcription suppressor to turn off the meristematic specific gene *KNOX1* to promote growth and differentiation (Hay et al., 2004; Piazza et al., 2005). In the process of establishing dorsal–ventral polarity in plant leaves, expression of *HD-ZIP III* and the MYB protein ASYMMETRIC LEAVES1 are the determinants of the ventral axis, while expression of *KANADI* (*KAN*), *ARF3*, and *ARF4* determine the fate of the dorsal axis. The *YABBY* gene acts downstream of the *KAN* gene in *A. thaliana* and is a decisive gene for leaf dorsal development. The function of *HD-ZIP III* genes in leaf polarization is relatively clear (Figure 2). The expression of *HD-ZIP III* was maintained only on the adaxial side, as members of *HD-ZIP III* family, are inhibited by miR165/166 on the abaxial side (Zhong and Ye, 2004). AGO1 is necessary for targeting miR165/166 to *HD-ZIP III* transcripts in leaves and is required for miR165/166 to regulate and restrict *PHBOLUSA* (*PHB*) to the adaxial side (Kidner and Martienssen, 2004). Like AGO1, the localization of AGO10 on the adaxial side of the leaf is necessary to inhibit the acellular autonomous miR165/166 activity and maintain the accumulation of *HD-ZIP III* mRNA in this region (Liu et al., 2009c).

At the same time, miR390 and its effector AGO7 are required to be involved in leaf polarization (Figure 2). TAS3 ta-siRNA determines the adaxial side by inhibiting the expression of *ARF3* and *ARF4* on the abaxial side of leaves (Chitwood et al., 2009). In *Zea maize* and *A. thaliana*, the ventral ta-siARF pathway interacts with the dorsal regulatory factors to some extent. Additionally, the ta-siARF pathway is also required to inhibit the expression of miR165/miR166, which allows for the proliferation of HD-ZIP III. Interference with ta-siARF pathway in maize will obviously affect leaf polarity. Wang et al. reported that miR396 also participated in leaf polarity formation by regulating the proliferation of leaf cells by targeting growth-regulating factors (GRFs), thus affecting the formation of dorsal–ventral axis polarity in leaves (Wang et al., 2011).

miRNAs also regulate leaf size and structure. The balance between miR396 and GRFs ultimately controls the number of cells in leaves and regulates the size of the meristem (Kim et al., 2003; Liu et al., 2009b; Rodriguez et al., 2010; Wang et al., 2011; Baucher et al., 2013; Debernardi et al., 2014). In addition, miR396 can also regulate leaf size through targeting *basic Helix–Loop–Helix 74* (Debernardi et al., 2012) and *CUC2*, which is necessary for the formation of the organ primordial boundary. miR319 regulates the growth and development of *A. thaliana* leaves by degrading the mRNA of the *TCP-like* transcription factor family which can regulate *CUC2* (Palatnik et al., 2007). In addition, *CUC2* expression is also regulated by the repressor miR164 (Koyama et al., 2010). The CUC2-miR164 system plays a key role in the evolution of composite leaves (Blein et al., 2008).

Meanwhile, miR319-TCP4 controls leaf senescence (Sun et al., 2017). The sequences of miR159 and miR319 are very similar, and the leaves of the miR159a miR159b double mutant are curled upward, indicating that miR159 also works on leaf development (Allen et al., 2007). miR393 and its target genes *TRANSPORT INHIBITOR RESPONSE 1* and *AUXIN SIGNALING F-BOX PROTEIN 1/2/3* can affect the shape and size of leaves by regulating the auxin response (Chen et al., 2011).

Stomata are special structures in the plant epidermis. miR824 and its target gene *Agamous Like 16* (*AGL16*) are involved in stomatal development. Overexpression of miR824 led to a decrease in stomatal density, similar to *agl16* mutant plants. However, when the regulation of miR824 on *AGL16* is destroyed, stomatal density will increase (Kutter et al., 2007). In maize, an increase of *GLl5* (*Glossyl5*) activity can increase the number of young leaves and delay the reproductive development. miRl72 can also promote the transformation from young leaves to mature leaves of maize through the negative regulation of *GLl5* mRNAs (Lauter et al., 2005). In tomato (*Solanum lycopersicum*), the *LANCEOLATE* gene encodes a *TCP* transcription factor. Its mutation or downregulation can cause compound leaves of plants to become single leaves. miR319 can target the *LANCEOLATE* (*LA*) gene and cause the formation of single leaves from multiple leaflets (Ori et al., 2007). Yanai et al. found that miR319 in tomato affects the differentiation and leaf shape by inhibiting the expression of the *SlGA20 oxidase1* gene, which is an enzyme involved in the GA synthesis pathway (Yanai et al., 2011). miR396 of the legume *Medicago truncatula* negatively regulates the expression of not only six *MtGRF* genes but also two *bHLH79*-like target genes and thus influences root growth and mycorrhizal associations (Bazin et al., 2013).

### The Role of miRNAs in Vascular Development

**Figure 3:**
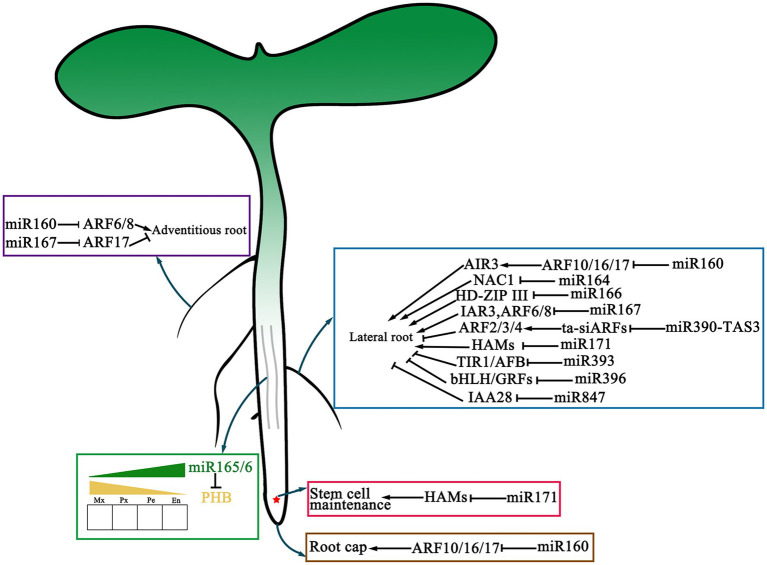
The function of miRNAs in the development of vascular and root. microRNAs are involved in vascular and root development. All of the mRNAs with verified functions in main root, lateral root, adventitious root development as well as their respective main targets are represented. The red asterisk represents the quiescent center (QC). The solid gray line in the middle of the main root represents the vascular tissue.

Vascular plants use xylem to transport water and nutrients absorbed by roots upward and the phloem to transport the carbohydrate assimilated by leaves downward. The vascular bundle consists of three neatly arranged tissues: xylem, procambium/cambium, and phloem (Figure 3). In *A. thaliana*, the *HD-ZIP III* gene family is strongly expressed in vascular bundles of roots, stems, and leaves. Overexpression of miR165 in *A. thaliana* can reduce the transcription level of all members of the *HD-ZIP III* family, thus regulating the polar differentiation of vascular tissue cells and affecting plant morphogenesis (Zhong and Ye, 1999; Kang and Dengler, 2002; Zhou et al., 2007; Muraro et al., 2014; Du and Wang, 2015; Jia et al., 2015). It was reported that miR166 controls the development of vascular cells and phloem cells by regulating the Homeobox 15 protein (*ATHB15*) in *A. thaliana* (Kim et al., 2005). In almost all plant species, it is found that the target site of miR165/166 in class *HD-ZIP III* genes is highly conserved suggesting that this module is necessary in plant development and evolution (Floyd and Bowman, 2004).

Some miRNAs are also related to cell wall synthesis and fiber development in plants (Kim, 2005). It has been reported that a new miRNA (miR857), is decisive in the formation of secondary walls of vascular in a copper ion-dependent manner. miR857 regulates the expression of the putative laccase *LACCASE7*, a member of laccase family of genes, at transcriptional level and affects lignin content (Zhao et al., 2015). A recent study highlighted that some components related to leaf polarity and vascular development, such as miR390, TAS3, and ARF, are conserved across all terrestrial plants. For example, in liverworts, TAS3 ta-siRNA targets ARF as it does in angiosperms (Xia et al., 2017). In *Nicotiana tabacum*, the semi-dominant *phv* (*phavoluta*) mutant without miRl65 regulation has abnormal radial growth of stem and leaf vascular systems, and the vascular tissue of stem nodes is discontinuous, showing that miRl65 controls the growth of vascular cambium suggesting that the function of miR165/166 in vascular development is also conserved in plants (Yu et al., 2005).

### The Role of miRNAs in Flower Development

Flower development is divided into three stages: flowering induction, flowering initiation, and floral organ development. It is a very complicated process involving multiple genes and is also an important event in development of higher plants. Many studies have shown that miRNA plays an important role in flowering.

**Figure 4:**
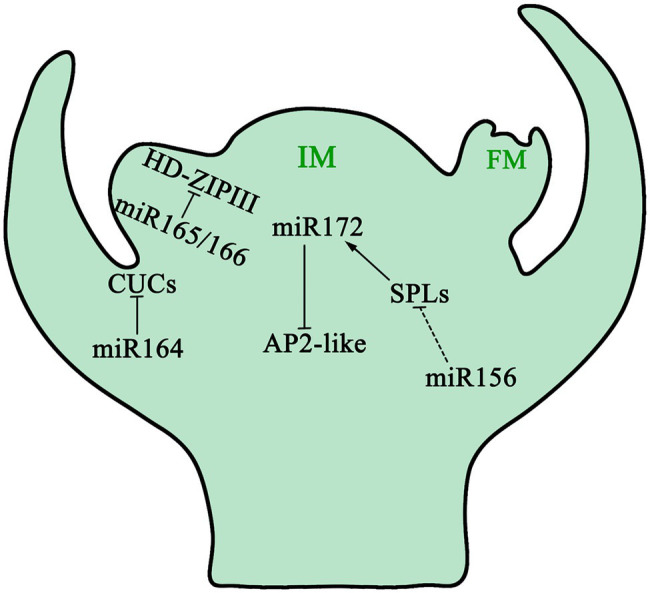
The function of miRNAs in inflorescence meristem. As plants change growing phases from juveniles to adults, downregulation of miR156 dampens the inhibition of *SPL* expression, which in turn promotes miR172 transcription. miR172 triggers the development of inflorescence meristem by reducing the mRNA level of *AP2-like* genes. Spatiotemporal functions of miR165/166 and their targets *HD-ZIP III* genes, together with miR164, restrict the functions of *CUCs* in specific regions of the boundary to maintain the inflorescence meristem. miR156 decreases during IM development, whereas miR172 increases. IM: Inflorescence Meristem; FM: Floral Meristem.

In *A. thaliana*, the vegetative phase transition is promoted by a group of plant-specific transcription factors (SBP/SPL proteins). Their expression is inhibited by miR156 and miR157 in the juvenile developmental stage. When the level of miR156/miR157 decreases, the abundance of SBP/SPL proteins increases and the plant changes from vegetative phase to reproductive phase (Xu et al., 2016a; He et al., 2018; Fouracre et al., 2021). miR156 is the main regulatory gene for plant growth cycle transformation, which affects plant phase transformation by targeting *SPL* (*Squamosa Promoter binding protein-Like*) transcription factors (He et al., 2018; Figure 4). Overexpression of miR156 and subsequent downregulation of *SPL3/5* resulted in delayed flowering period of *A. thaliana*; downregulation of *SPL9* and *SPL15* resulted in shortened leaf plastochrons, slower growth, and extremely abundant leaves of *A. thaliana* (Schwab et al., 2005; Wu and Poethig, 2006; Xu et al., 2016b; Zhang et al., 2019). The role of miR156 and SPLs in flower development was also reported in rice (Xie et al., 2006). Studies have shown that the fine negative regulation of miR156 on *SPL3* ultimately affects the flowering phase transition process of *A. thaliana* by changing the expression of the *FT* gene in *A. thaliana* leaves leading to delayed flowering (Kim et al., 2012). Similar to the function of juvenile hormones in insects, high concentrations of miR156 keeps plants in the juvenile developmental stage. As development progresses, the amount of miR156 decreases gradually, which promotes the juvenile-to-adult transition. Further studies showed that the decrease of miR156 content was not related to the absolute age (i.e., absolute time) of plants, but associated with the physiological age of plants (Cheng et al., 2021b).

miR172 is similar to miR156: namely, both are involved in controlling flowering time and the formation of floral organs by degrading and inhibiting target mRNA (Jung et al., 2007). miR172 regulates the transformation of plant development from the juvenile to flowering stage by regulating AP2-like genes including *SM-LIKE 2*, *SCHNARCHZAPFEN*, and *TARGET OF EARLY ACTIVATION TAGGED 1/2/3*. miR172 regulates plant flowering time, flower organ determination, flower morphogenesis, and plant development by controlling *AP2* transcription factors (Aukerman and Sakai, 2003). Overexpression of miR172 in *A. thaliana* will promote early flowering, while overexpression of *AP2* genes will delay flowering.

In addition, miR156 and miR172 interact together in some parts of the plant growth cycle that are regulated by miRNA. miR156 inhibited the expression of the *SPL* family, while some SPLs promoted the expression of miR172. Previous studies have shown that the miR156-SPL-miR172 pathway in *A. thaliana* is the decisive factor in controlling the juvenile-to-adult transition. The miR156-SPL-miR172 pathway can be divided into two modules: the leaf module and the apical meristem module, both of which have different combinations of SPL and miR172 encoding gene modules. In leaves, the SPL9-miR172b/c modules regulate flowering time by regulating the expression of the *FT* gene; while in apical meristem, the SPL15-miR172d modulus promotes flowering by activating the expression of *MADS-box* genes. In addition, the expression of the *MIR172* gene can be regulated by ambient temperature and photoperiod, and different *MIR172* genes have different response patterns (Lian et al., 2021).

Other miRNAs, such as miR159 and miR319, also function in flowering development. Their target genes are *MYB* and *TCP* (*TCP FAMILY TRANSCRIPTION FACTOR*) transcription factors, respectively. Overexpression of miR159 and miR319 will cause floral development disorders, such as delayed flowering (Palatnik et al., 2007). miR159 can regulate the expression of *MYB33* and *MYB65*, and a loss-of-function miR159 displays strong pleiotropic defects, stunted growth, curled leaves, defective sepals, petals, and anthers in *A. thaliana* (Achard et al., 2004; Millar and Gubler, 2005; Tsuji et al., 2006; Yu et al., 2012). At the same time, miR159 can prevent the over-activation of miR156, thus regulating the phase transition of *A. thaliana* in vegetative developmental period (Guo et al., 2017). *MYB33*, the target of miR159, promotes the transcription of *ABA INSENSITIVE 5* (*ABI5*) by binding directly to its promoter, then *ABI5* plays a role in the upstream of miR156 and regulates the juvenile-to-adult transition in *Arabidopsis* by affecting the gene expression in the miR156-SPL pathway (Guo et al., 2021).

In *A. thaliana*, miR164 regulates the number of petals and the differentiation of floral organ marginal cells and apical meristem cells by increasing the accumulation of CUC transcription factors in the boundary. Meanwhile, overexpression of miR164 leads to sepal fusion and reduction of petal number, suggesting that miR164 is related to the activity of flower meristem and the specific boundary division of the meristem region (Laufs et al., 2004; Jung et al., 2009).

miR165/166 also regulates flower morphogenesis. miR166/165 gene showed tissue-specific expression patterns in different flower organs. miR166a was mainly expressed in stamens, while miR166b was highly expressed in ovule and stigma. miR166d and miR165a were highly expressed in ovule. In contrast, miR166g had a broad expression in the stigma, stamen, and receptacle, but not in the ovule (Jung and Park, 2007). In terms of meristem activity regulation, miR165/166 is closely related to meristem formation in floral organs (Zhang et al., 2007). In the *Arabidopsis* mutants with miR165/166 overexpression, the flower structure was seriously damaged. For example, when miR166 is overproduced in *mum enhancer 1* and *jabba* mutants, and the pistil population is very small and the number of carpels is also reduced.

The significant increase of miR396 expression can cause the bending of the stigma in flowers, which demonstrated that miR396 also participates in the regulation of flower development. In *A. thaliana*, excessive production of miR167 displays floral defects resulting to that filaments were abnormally short, anthers could not properly release pollen, and pollen grains did not germinate (Ru et al., 2006). *ARF6* and *ARF8*, the target genes of miR167, play a meaningful role in the regulation of pistil and stamen population. miR167 also controls the fertility of male and female flowers of *A. thaliana* (Wu et al., 2006).

In addition to regulating reproductive organ morphology in the model organism *A. thaliana,* miRNAs have also been shown to regulate these organs in other plants. Tomato miR156b performs a key role in controlling flower and fruit morphology by regulating meristem activity and the initial stage of fruit development. Also, in tomato, overexpression of *A, thaliana* miR167a causes the downregulation of *ARF6* and *ARF8*, resulting in serious disorders in floral organ development and female gamete fertility (Liu et al., 2014b). In *Petunia* and *Antirrhinum* species, researchers found that miR169 can partially replace AP2, which results from the fact miR169 can regulate transcription factor *NF-YA*, thus affecting the development of flower organs (Chen, 2004; Cartolano et al., 2007; Zhao et al., 2009; Waheed and Zeng, 2020).

miRNAs also regulate flower and seed production in monocots. In rice, Zhu et al. found that overexpression of miR172 can cause spikelet deletion, floral organ development malformation, and fertility reduction (Zhu et al., 2009). OsmiR397 is a miRNA that is expressed at a high level in the young panicles and grains of rice, which increases grain yield by downregulating its target gene *OsLAC*. Overexpression of OsmiR397 can increase grain size and promote panicle branching (Zhang et al., 2013). In maize, Chuck et al. showed that miRNA-targeting SBP-box transcription factor *tasselsheath4* plays a critical role in the development of maize bracts and the establishment of meristem boundaries in inflorescences (Chuck et al., 2010, 2014) auxin.

### Other Processes Involving miRNAs

miRNA also plays an essential regulatory role in other developmental processes. In *A. thaliana*, auxin response factors *ARF10*, *ARF16*, and *ARF17* are targeted by miR160. Studies have shown that miR160 plays a very important role in the negative regulation of *ARF10* to promote seed germination (Liu et al., 2007). Llave et al. found that during *Arabidopsis* root growth, root cap cell formation is related to miR160, which controls stem cell differentiation at the end of the root meristematic region and determines root growth direction by regulating the expression of *ARF10* (Figure 3; Llave et al., 2002).

In addition, miR164 and miR390 greatly influence the development of plant root organs, including root cap formation, lateral root development, and adventitious root formation (Yoon et al., 2010). The process of lateral root growth of *A. thaliana* is regulated by miR164. Guo et al. found that miR164 can mediate *NAC1* expression after being induced by auxin, thus affecting auxin transmission and regulating lateral root growth (Figure 3; Guo et al., 2005).

miR165/166 is related to the formation of xylem and cell arrangement in plants. The regulation of miRNA on plant tissue development is a complex molecular process (Figure 3; Carlsbecker et al., 2010). The same miRNA may have the multiple functions in different tissues. For example, miR165/miR166 is also related to leaf polarization in addition to xylem and cell arrangement as mentioned in a previous section (Tatematsu et al., 2015; Manuela and Xu, 2020).

Furthermore, miRNA is involved in regulating plant morphological structure and yield, which is important in crop plants. In soybean, the miR156-SPL gene module plays a key role in regulating the morphological structure and yield of soybean. In transgenic soybean overexpressing miR156b, axillary bud formation and branching are regulated by reducing the expression amount of *SPL9d* (Wang and Wang, 2015). In rice, inhibiting the expression of miR1432 or overexpressing *OsACOT* (*Acyl-CoA Thioesterase*) can cause the grain weight to be significantly boosted by increasing the grain filling rate, which can improve crop yield (Zhao et al., 2019). Genetic analysis shows that *OsSPL7* is the target of miR156f, which regulates plant morphological structure, namely tillering and height of rice (Dai et al., 2018). At the same time, *OsSPL7* directly binds to the *OsGH3.8* promoter to regulate its transcription, indicating that the miR156f-OsSPL7-OsGH3.8 is the complete regulatory pathway for these traits in rice.

miRNA is widely connected to plant diseases and environmental stress responses. Virus infections can greatly influence plant morphology and productivity. More and more evidence has shown that miRNA is related to virus-mediated diseases and virus-induced gene silencing (Chapman et al., 2004). More than 30 RNA silencing suppressors, also known as pathogenic factors, have been identified from plant viruses, including p19, p21, p25, and p69. Pathogenic factors can usually hinder the formation of siRNA, affect the stability of siRNA, or interfere with the combination of siRNA and RISC complexes, and can also lead to the generation of other diseases in plants and cause developmental malformation. Excessive HC-Pro protease (helper-component proteinase) in plants will reduce miR171 level and produce developmental deletion plants associated with miR171 which included branching defects, an increased number of short vegetative phytomers and late flowering. Through the overexpression of the *Hc-Pro* gene in *A. thaliana*, it was found that most miR171 target mRNAs are increased which results in virus-mediated diseases in plants (Kasschau et al., 2003).

Under abiotic stress, plants can directly synthesize some miRNAs and induce low or excessive expression of other miRNAs. These miRNAs act on transcription factors related to stress resistance, *Plant Growth Regulator 9* response protein genes, stress tolerance protein genes, and other target genes, which enables plants to quickly respond to environmental changes. In plants, miRNA responding to stress was first found in *A. thaliana* (Jones-Rhoades and Bartel, 2004). The expression of miR393 in *A. thaliana* was significantly upregulated after low temperature, drought, salt, or hormone (ABA) treatment. However, no responses to drought or NaCl were observed when miR310 and miR319 were upregulated after low-temperature stress indicating that these two miRNAs only function in low-temperature response. miR389a was downregulated after the above stress was induced (Sunkar and Zhu, 2004). miR393, miR397, miR402 (Sunkar and Zhu, 2004), miR165/miR166, miR169, and miR172 (Zhou et al., 2008) were all found to be induced by low temperature to enhance the plant resistance. In *A. thaliana*, the expression level of miR395 increased in the absence of sulfate, while the expression level of miR399 was upregulated, and the mRNA level of its target gene *PHO2/UBC24* (*PHOSPHATE 2*) was lower (Chiou et al., 2006). miR169 is downregulated in a drought environment. Compared to wild-type plants, plants overexpressing miR169a or plants with *Nuclear transcription factor Y subunit A-5* deletion of miR169’s target gene are more likely to lose leaf water and are more sensitive to drought (Li et al., 2008). In *grapevine*, miR398 participates in plant biotic stress, heavy metals, high salt, drought, ultraviolet radiation, and other abiotic stresses through the targeted regulation of two superoxide dismutases, *COPPER/ZINC SUPEROXIDE DISMUTASE 1/2* (Leng et al., 2017).

As mentioned above, studies in *Arabidopsis thaliana* and other plants have showed that miRNAs participate in many biological processes. Compared with the plant-wide action of hormones, miRNAs are crucial in precise regulation of gene expression in a tissue-specific pattern. How the plants integrate miRNAs fine regulation into hormonal system pathway to modulate tissue formation deserves more attention. Study the role and mechanism of miRNA movement between cells and tissues are vital to understand miRNA function.

## The Interaction Between miRNAs and Plant Hormones

Plant hormones are important regulatory factors synthesized in plants. They regulate plant growth, development, and differentiation either individually or together. Plant hormones mainly include auxin (AUX), cytokinin (CK), abscisic acid (ABA), gibberellic acid (GA), ethylene (ET), brassinosteroid (BR), and jasmonic acid (JA). As signaling molecules regulating plant growth and development, these hormones have absolutely necessary function in controlling development timing, metabolism, and stress response through the whole plant growth cycle. Specific stages of development often involve the participation of multiple hormones; this enables plant cells to respond adaptively to development signals and changes in their internal and external environment (Li et al., 2020). miRNAs coordinate with hormones by negatively regulating target genes in hormonal pathways. It was found that in the seedlings, the overall miRNA accumulation level decreased after *HYL1* mutation, which displayed a variety of developmental defect phenotypes and abnormal sensitivity to ABA, AUX, and CK, indicating that miRNA is related to the signal responses of these hormones (Han et al., 2004). Many miRNA gene promoters contain hormone response elements as well as cis-elements response to stresses, indicating that the regulation of miRNA gene transcription may be a way of hormone and stress response (Ding et al., 2013).

miRNAs regulate auxin receptors and several transcription factors in plants. In *Arabidopsis*, when the expression of miR160 was silenced, the expression levels of *ARF16* and *ARF17* genes increased, which led to abnormal germ development, cotyledon shape defect, slow inflorescence development, stamen reduction, root shortening, and other adverse developmental symptoms. However, overexpression of miR160 in *Arabidopsis* inhibited the development of root cap and increased the number of lateral roots (Figure 3; Mallory et al., 2005; Wang et al., 2005). These results indicate that precise accumulation of miR160 is crucial to auxin-related plant development. miR167 and ARF6/8 co-regulate adventitious root formation (Gutierrez et al., 2009). miR847 targets and silences IAA28, the AUX/IAA inhibitory protein, to activate the auxin signaling pathway. The ubiquitination-mediated degradation of the IAA28 protein combined with miR847/IAA28 mRNA regulatory module to achieve the rapid disinhibition of the auxin signaling pathway (Wang and Guo, 2015). At the same time, miR165/166 directly targets *PHB*, an activator of *ARF5*, and then triggers the expression of miR390, which directly lead to the accumulation of ta-siRNAs (tasiR-ARF3/4; Marin et al., 2010; Muller et al., 2016; Dastidar et al., 2019). In addition, the miR165/166-tasiR-ARFs module also establishes the paraxial/distal polarity of the blade.

miR159 and miR319 inhibit the expression of *SHOOTMERISTEMLESS* and *BREVIPEDICELLUS*, and then enhance the expression of *IPT* (*ISOPENTENYL TRANSFERASE*) and promote the biosynthesis of CK in SAM (Rubio-Somoza and Weigel, 2013; Scofield et al., 2014). At the cytokinin signal transduction level, the miR156-SPL9 complex modulates cytokinin-related plant regeneration by inhibiting the *B-type ARR genes9* [*type B Arabidopsis Response Regulators* (*ARRs*)], which are transcription factors that act as positive regulators in the two-component cytokinin signaling pathway (Zhang et al., 2015).

miRNAs also affect the biosynthesis and signal transduction of cytokinin through auxin, and then continue to maintain the dynamic balance between auxin and cytokinin, such as miR160 and miR165/6 (Dello et al., 2012; Liu et al., 2016). Another signaling molecule, gibberellin, can regulate the levels of various miRNAs through DELLA (aspartic acid–glutamic acid–leucine–leucine–alanine) protein and its interacting proteins, such as IDD2 (indeterminate (ID)-domain 2), PHYTOCHROME-INTERACTING FACTOR 4, or SCARECROW-LIKE (SCL; Han et al., 2014; Fan et al., 2018). Conversely, miRNAs can directly regulate GA biosynthesis and signal transduction through different complexes such as miR156-SPL, miR171-SCL, and miR159-GAMYB(L)s modules (Yu et al., 2012; Ma et al., 2014; Sun et al., 2018; Millar et al., 2019). Brassinosteroids (BR) negatively regulate miRNA-mediated translation inhibition of target genes by interfering with the distribution and localization pattern of AGO1, the miRNA effector protein, in the endoplasmic reticulum (Wang et al., 2021d).

miRNAs can regulate seed germination and leaf senescence by affecting the levels of ABA and ethylene. ABA, the signaling hormone, and SnRK2 (SNF1-related protein kinase 2) protein kinase, the core component of the osmotic stress response pathway, can regulate miRNA synthesis (Yan et al., 2017). At the same time, the ABA and ethylene signaling pathway can cause feedback on the level of sRNA by affecting the core protein in sRNA synthesis pathway, such as CBP20 (CAP-BINDING PROTEIN 20; Kim et al., 2008; Li et al., 2012, 2013; Zhang et al., 2016). Therefore, miRNAs coordinate with hormone responses in many ways and play an important role in plant development.

## Role of Small RNA Movement in Plant Development

Plant small RNAs can spread silencing signals by moving in plants to participate in plant development regulation and respond to environmental stresses. Usually, mobile small RNAs generate sharply defined domains of target gene expression through an intrinsic and direct threshold-based readouts of their mobility gradients to drive developmental patterning (Skopelitis et al., 2017). There are two main types of small RNA movement in plants: one is short-distance (cell-to-cell) movement between neighboring cells, the other is long-distance (such as shoot to root or root to shoot) movement in plants. Currently, there is a hypothesis that 21 nt-siRNA are mainly involved in short-distance transport and 23\u201324 nt small RNAs are mainly involved in long-distance transport. The mechanisms of these two types of small RNA movement may be different (Tamiru et al., 2018), and will be explored in the following sections.

### Short-Distance Movement

The short-distance movement of plant small RNAs is was thought to be mainly conferred *via* plasmodesmata between adjacent cells (Vaten et al., 2011). However, using a type of miR-GFP sensor system, it has been found that small RNAs are an independent mobile unit, and their mechanisms of movement between cells are different from that of proteins (Skopelitis et al., 2018). Some small RNAs have been discovered that can move in short distances of up to ten files of cells. For example, mature miR165/166 can move from the endoderm of the root to the vasculature, thereby forming a gradient-like distribution of miR165/166 to regulate the expression pattern of its target gene *PHB* and finally complete the establishment of proto- and metaxylem (Carlsbecker et al., 2010). In leaves, miR165/166 can be moved from the abaxial surface to the adaxial side, also forming a gradient to regulate the expression pattern of *HD-ZIP III* genes and ultimately form leaf polarity. In the SAM, miR394 moves to the cells in the L2 and L3 layers to repress its target gene *LCR* as a mobile signal produced by L1 layer cell. Repression of *LCR* signal in the underneath stem cells is used to maintain stem cell pluripotency by influencing the WUS-CLV loop (Knauer et al., 2013). In addition to miRNAs, PhasiRNAs have also been found to be able to move from cell to cell. For example, tasiR-ARF is produced from long non-coding RNAs transcribed at the TAS3 loci by the processing of the miR390-AGO7 complex on the adaxial side of leaves (Allen et al., 2005; Endo et al., 2013). These tasiR-ARFs can move to the abaxial side of leaves and form a gradient of to inhibit the expression of *ARF3* on adaxial side. Inhibition of *ARF3* expression ensures the establishment of leaf polarity patterns (Chitwood et al., 2009). Recent experiments show that processed tasiR-ARFs in the apical epidermal cells can move to hypodermal cells in the nucellar region to repress *ARF3* expression and suppress ectopic megaspore mother cell (MMC) fate (Su et al., 2020).

### Long-Distance Movement

The long-distance movement of plant small RNA is mainly mediated through the phloem following source–sink relationships (Melnyk et al., 2011; Tamiru et al., 2018). In line with this, miRNAs have been found in the phloem saps of multiple plants (Tamiru et al., 2018). For example, miR172 was found in the vascular bundles of potatoes, indicating that miR172 might be mobile or that it regulates long-distance signals to induce tuberization(Marin et al., 2010). In *Brassica napus*, using small RNA sequencing, it was discovered that levels of miR395, miR398, and miR399 in the phloem are strongly increased in response to sulphate, copper, or phosphate starvation, respectively (Buhtz et al., 2008).

In *Arabidopsis*, miR399 moves from shoot to root to inhibit the expression of its target gene *PHO2* in response to phosphate homeostasis (Lin et al., 2008; Pant et al., 2008). During phosphate starvation, miR827 and miR2112a can also move from shoot to root (Huen et al., 2017). miR2112 can move from shoot to root to inhibit the expression of symbiosis suppressor TOO MUCH LOVE, thereby controlling rhizobial infection (Tsikou et al., 2018).

## Future Perspectives

Understanding the elaborate regulation of plant development by miRNAs is crucial for crop breeding. Knocking out dominant genes in development often causes lethality in plants, while miRNAs can safely modify gene expression to some extent and improve plant development. In rice, the number of branches (including tiller and inflorescence branches) determines grain yield. It was found that the genes regulating rice tillering and panicle branching consisted of miR156/miR529/SPL and miR172/AP2 modules. The *SPL* gene negatively controls tillering, but positively regulates the transformation of inflorescence meristem and spikelet. Changes in *SPL* expression will reduce panicle branching (Wang and Wang, 2015). In the regulation of seed size and grain yield, OsmiR397 can increase grain size, promote panicle branching, and increase grain yield by downregulating its target gene *OsLAC* (Zhang et al., 2013). miR1432-OsACOT modules are involved in fatty acid metabolism and plant hormone biosynthesis, and crucial for rice (Zhao et al., 2019). miR319s negatively affects tiller number and grain yield by targeting *OsTCP21* and *OsGAmyb* (Wang et al., 2021c). Changes of “miR168-AGO1” regulatory pathway influence several “miRNA-target gene” loops, which regulate the immunity and growth of rice, respectively. Among these, the “miR535-SPL14” loop regulates the yield and immunity of rice, the “miR164-NAC11” loop regulates the growth period and immunity of rice, and miR1320 regulates the immunity of rice (Wang et al., 2021a). In maize, *TASSELSEED4* encodes miR172 to control sex determination and meristem cell fate by targeting *IDS1* (*Indeterminate Spikelet1*). Moreover, miR156a-l acts on several SPL genes during the transition from young to mature ear, and indirectly activates miR172 through *SPLs* (Lauter et al., 2005; Chuck et al., 2007b; Salvi et al., 2007). In agriculture, epigenetic variations account for a great proportion for change in crop yield. SNPs located in non-coding regions are paid more and more attention by breeders in population genetic analysis and traditional hybrid breeding. New strategies such as Short Tandem Target Mimic (STTM), a specific miRNA targeting method which is effective in blocking small RNA functions in plants (Tang et al., 2012), are adapted and utilized in generating transgenic crops. As *MIR* genes are usually short, EMS mutation and T-DNA inserted mutation are difficult to achieve ideal mutants for *MIR* genes. However, the advances of genome editing technologies make modification of miRNA expression to increase crop yield become easier.

Modes of miRNA function need to be further explored. miRNAs also act as environmental response factors, endowing plants with corresponding phenotypes and promoting plant evolution and adaptation. For example, the essential role of HD-ZIP III-miR165/166 signaling pathway in meristematic tissues and the dual regulatory role of miR156/miR172 in flower determination are conserved in plant kingdom. The function of miRNAs and their specific mechanisms need to be further studied. It is still not clearly understood how miRNAs specifically regulate a biological process in certain temporal and spatial patterns. Many miRNA gene promoters contain plant hormones and cis-elements of stress response, indicating that regulation of miRNA gene transcription may be a way to respond to plant hormone and stresses. The expression of *AGO10* is precisely regulated by auxin, brassinolide, and light to initiate axillary meristem in certain leaf axils. This provides a way to modify gene expression in a tissue-specific pattern and potentiate modulation of organ development at certain stages.

Recently, great importance has been attached to small RNA movement between cells, tissues as well as organisms by plant researchers. Much effort is made to uncover the role and mechanism of small RNA movement. So far, it is evidenced that miRNA can move to form gradient distribution between different tissues. After biogenesis, miRNA is protected from degradation and is transported to destination cells. It is noteworthy that miRNA needs to reach a certain threshold level before it can function in a non-cellular autonomous way. How intermediate steps influence miRNA movement and its non-cellular autonomous function need more studies. To understand and prime plants for abiotic stresses, it is also worth further studies to elaborate the correlation between hormone concentration and miRNA movement.

In addition, biotic and abiotic stresses can induce plants to produce new sRNA. For example, *A. thaliana* can produce a large number of 22 nt siRNAs dependent on DCL2 and RDR6 under stresses such as nitrogen deficiency. However, it is still a puzzle as to why only a small number of gene loci in *A. thaliana* can produce 22 nt siRNAs. Meanwhile, there is also a big gap in knowledge of the synthesis of 22 nt siRNAs to their biological function. More evidence is needed to verify whether 22 nt siRNAs can also regulate target genes in distal organs due to the cellular non-autonomy of sRNA. Therefore, the improvement of sequencing technology and miRNA research methods are highly recommended here. With the help of various single-cell omics and nanopore sequencing, more miRNAs, their action mechanisms, and their regulatory pathways will be discovered in model plants, which will provide important theoretical basis for understanding how miRNA regulates plant growth and development and can then be applied to agriculturally important plants.

## Author Contributions

QD, BH, and CZ wrote the article. All authors read and approved the manuscript.

## Funding

Research in the Zhang lab is supported by the Strategic Priority Research Program of the Chinese Academy of Sciences, Grant Nos. XDA26030200 and XDA24010106-2 to CZ.

## Conflict of Interest

The authors declare that the research was conducted in the absence of any commercial or financial relationships that could be construed as a potential conflict of interest.

## Publisher’s Note

All claims expressed in this article are solely those of the authors and do not necessarily represent those of their affiliated organizations, or those of the publisher, the editors and the reviewers. Any product that may be evaluated in this article, or claim that may be made by its manufacturer, is not guaranteed or endorsed by the publisher.

## References

[ref1] Abdel-Ghany S. E. Pilon M. (2008). MicroRNA-mediated systemic down-regulation of copper protein expression in response to low copper availability in Arabidopsis. J. Biol. Chem. 283, 15932–15945. doi: 10.1074/jbc.M801406200, PMID: 18408011 PMC3259626

[ref2] Achard P. Herr A. Baulcombe D. C. Harberd N. P. (2004). Modulation of floral development by a gibberellin-regulated microRNA. Development 131, 3357–3365. doi: 10.1242/dev.01206, PMID: 15226253

[ref3] Allen R. S. Li J. Stahle M. I. Dubroue A. Gubler F. (2007). Genetic analysis reveals functional redundancy and the major target genes of the Arabidopsis miR159 family. Proc. Natl. Acad. Sci. U. S. A. 104, 16371–16376. doi: 10.1073/pnas.0707653104 17916625 PMC2042213

[ref4] Allen E. Xie Z. Gustafson A. M. Carrington J. C. (2005). MicroRNA-directed phasing during trans-acting siRNA biogenesis in plants. Cell 121, 207–221. doi: 10.1016/j.cell.2005.04.004, PMID: 15851028

[ref5] Arikit S. Xia R. Kakrana A. Huang K. Zhai J. (2014). An atlas of soybean small RNAs identifies phased siRNAs from hundreds of coding genes. Plant Cell 26, 4584–4601. doi: 10.1105/tpc.114.131847 25465409 PMC4311202

[ref6] Aukerman M. J. Sakai H. (2003). Regulation of flowering time and floral organ identity by a MicroRNA and its APETALA2-like target genes. Plant Cell 15, 2730–2741. doi: 10.1105/tpc.016238, PMID: 14555699 PMC280575

[ref7] Baucher M. Moussawi J. Vandeputte O. M. Monteyne D. Mol A. (2013). A role for the miR396/GRF network in specification of organ type during flower development, as supported by ectopic expression of Populus trichocarpa miR396c in transgenic tobacco. Plant Biol. 15, 892–898. doi: 10.1111/j.1438-8677.2012.00696.x, PMID: 23173976

[ref8] Baumann K. (2013). Plant cell biology: Mobile miRNAs for stem cell maintenance. Nat. Rev. Mol. Cell Biol. 14:128. doi: 10.1038/nrm3529, PMID: 23385721

[ref9] Baumberger N. Baulcombe D. C. (2005). Arabidopsis ARGONAUTE1 is an RNA slicer that selectively recruits microRNAs and short interfering RNAs. Proc. Natl. Acad. Sci. U. S. A. 102, 11928–11933. doi: 10.1073/pnas.0505461102 16081530 PMC1182554

[ref10] Bazin J. Khan G. A. Combier J. P. Bustos-Sanmamed P. Debernardi J. M. (2013). MiR396 affects mycorrhization and root meristem activity in the legume Medicago truncatula. Plant J. 74, 920–934. doi: 10.1111/tpj.12178, PMID: 23566016

[ref11] Bergmann D. C. Sack F. D. (2007). Stomatal development. Annu. Rev. Plant Biol. 58, 163–181. doi: 10.1146/annurev.arplant.58.032806.104023, PMID: 17201685

[ref12] Blein T. Pulido A. Vialette-Guiraud A. Nikovics K. Morin H. (2008). A conserved molecular framework for compound leaf development. Science 322, 1835–1839. doi: 10.1126/science.1166168, PMID: 19095941

[ref13] Bologna N. G. Iselin R. Abriata L. A. Sarazin A. Pumplin N. (2018). Nucleo-cytosolic shuttling of ARGONAUTE1 prompts a revised model of the plant MicroRNA pathway. Mol. Cell 69, 709–719. doi: 10.1016/j.molcel.2018.01.007, PMID: 29398448

[ref14] Borges F. Martienssen R. A. (2015). The expanding world of small RNAs in plants. Nat. Rev. Mol. Cell Biol. 16, 727–741. doi: 10.1038/nrm4085, PMID: 26530390 PMC4948178

[ref15] Bresso E. G. Chorostecki U. Rodriguez R. E. Palatnik J. F. Schommer C. (2018). Spatial control of gene expression by miR319-regulated TCP transcription factors in leaf development. Plant Physiol. 176, 1694–1708. doi: 10.1104/pp.17.00823, PMID: 29133375 PMC5813565

[ref16] Brioudes F. Jay F. Sarazin A. Grentzinger T. Devers E. A. (2021). HASTY, the Arabidopsis EXPORTIN5 ortholog, regulates cell-to-cell and vascular microRNA movement. EMBO J. 40:e107455. doi: 10.15252/embj.2020107455, PMID: 34152631 PMC8327949

[ref17] Brodersen P. Sakvarelidze-Achard L. Bruun-Rasmussen M. Dunoyer P. Yamamoto Y. Y. (2008). Widespread translational inhibition by plant miRNAs and siRNAs. Science 320, 1185–1190. doi: 10.1126/science.1159151, PMID: 18483398

[ref18] Buhtz A. Springer F. Chappell L. Baulcombe D. C. Kehr J. (2008). Identification and characterization of small RNAs from the phloem of Brassica napus. Plant J. 53, 739–749. doi: 10.1111/j.1365-313X.2007.03368.x, PMID: 18005229

[ref19] Bustos-Sanmamed P. Mao G. Deng Y. Elouet M. Khan G. A. (2013). Overexpression of miR160 affects root growth and nitrogen-fixing nodule number in Medicago truncatula. Funct. Plant Biol. 40, 1208–1220. doi: 10.1071/FP13123, PMID: 32481189

[ref20] Cambiagno D. A. Giudicatti A. J. Arce A. L. Gagliardi D. Li L. (2021). HASTY modulates miRNA biogenesis by linking pri-miRNA transcription and processing. Mol. Plant 14, 426–439. doi: 10.1016/j.molp.2020.12.019, PMID: 33385584

[ref21] Carbonell A. Fahlgren N. Garcia-Ruiz H. Gilbert K. B. Montgomery T. A. (2012). Functional analysis of three Arabidopsis ARGONAUTES using slicer-defective mutants. Plant Cell 24, 3613–3629. doi: 10.1105/tpc.112.099945, PMID: 23023169 PMC3480291

[ref22] Carlsbecker A. Lee J. Y. Roberts C. J. Dettmer J. Lehesranta S. (2010). Cell signalling by microRNA165/6 directs gene dose-dependent root cell fate. Nature 465, 316–321. doi: 10.1038/nature08977, PMID: 20410882 PMC2967782

[ref23] Cartolano M. Castillo R. Efremova N. Kuckenberg M. Zethof J. (2007). A conserved microRNA module exerts homeotic control over Petunia hybrida and Antirrhinum majus floral organ identity. Nat. Genet. 39, 901–905. doi: 10.1038/ng2056 17589508

[ref24] Caruana J. C. Dhar N. Raina R. (2020). Overexpression of Arabidopsis microRNA167 induces salicylic acid-dependent defense against pseudomonas syringae through the regulation of its targets ARF6 and ARF8. Plant Direct 4:e270. doi: 10.1002/pld3.270, PMID: 33005858 PMC7510475

[ref25] Chandran V. Wang H. Gao F. Cao X. L. Chen Y. P. (2018). MiR396-OsGRFs module balances growth and rice blast disease-resistance. Front. Plant Sci. 9:1999. doi: 10.3389/fpls.2018.01999, PMID: 30693011 PMC6339958

[ref26] Chapman E. J. Prokhnevsky A. I. Gopinath K. Dolja V. V. Carrington J. C. (2004). Viral RNA silencing suppressors inhibit the microRNA pathway at an intermediate step. Genes Dev. 18, 1179–1186. doi: 10.1101/gad.1201204, PMID: 15131083 PMC415642

[ref27] Chen X. (2004). A microRNA as a translational repressor of APETALA2 in Arabidopsis flower development. Science 303, 2022–2025. doi: 10.1126/science.1088060, PMID: 12893888 PMC5127708

[ref28] Chen Z. H. Bao M. L. Sun Y. Z. Yang Y. J. Xu X. H. (2011). Regulation of auxin response by miR393-targeted transport inhibitor response protein 1 is involved in normal development in Arabidopsis. Plant Mol. Biol. 77, 619–629. doi: 10.1007/s11103-011-9838-1, PMID: 22042293

[ref29] Chen H. M. Chen L. T. Patel K. Li Y. H. Baulcombe D. C. (2010). 22-nucleotide RNAs trigger secondary siRNA biogenesis in plants. Proc. Natl. Acad. Sci. U. S. A. 107, 15269–15274. doi: 10.1073/pnas.1001738107 20643946 PMC2930544

[ref30] Chen X. Rechavi O. (2021). Plant and animal small RNA communications between cells and organisms. Nat. Rev. Mol. Cell Biol. doi: 10.1038/s41580-021-00425-y [Epub ahead of print]. PMC9208737 34707241

[ref31] Cheng X. He Q. Tang S. Wang H. Zhang X. (2021a). The miR172/IDS1 signaling module confers salt tolerance through maintaining ROS homeostasis in cereal crops. New Phytol. 230, 1017–1033. doi: 10.1111/nph.17211, PMID: 33462818

[ref32] Cheng Y. J. Shang G. D. Xu Z. G. Yu S. Wu L. Y. (2021b). Cell division in the shoot apical meristem is a trigger for miR156 decline and vegetative phase transition in Arabidopsis. Proc. Natl. Acad. Sci. U. S. A. 118:e2115667118. doi: 10.1073/pnas.2115667118, PMID: 34750273 PMC8609562

[ref33] Chiou T. J. Aung K. Lin S. I. Wu C. C. Chiang S. F. (2006). Regulation of phosphate homeostasis by MicroRNA in Arabidopsis. Plant Cell 18, 412–421. doi: 10.1105/tpc.105.038943, PMID: 16387831 PMC1356548

[ref34] Chitwood D. H. Nogueira F. T. Howell M. D. Montgomery T. A. (2009). Pattern formation *via* small RNA mobility. Genes Dev. 23, 549–554. doi: 10.1101/gad.1770009, PMID: 19270155 PMC2658522

[ref35] Chuck G. S. Brown P. J. Meeley R. Hake S. (2014). Maize SBP-box transcription factors unbranched2 and unbranched3 affect yield traits by regulating the rate of lateral primordia initiation. Proc. Natl. Acad. Sci. U. S. A. 111, 18775–18780. doi: 10.1073/pnas.1407401112, PMID: 25512525 PMC4284592

[ref36] Chuck G. Cigan A. M. Saeteurn K. Hake S. (2007a). The heterochronic maize mutant Corngrass1 results from overexpression of a tandem microRNA. Nat. Genet. 39, 544–549. doi: 10.1038/ng2001, PMID: 17369828

[ref37] Chuck G. Meeley R. Irish E. Sakai H. Hake S. (2007b). The maize tasselseed4 microRNA controls sex determination and meristem cell fate by targeting Tasselseed6/indeterminate spikelet1. Nat. Genet. 39, 1517–1521. doi: 10.1038/ng.2007.20, PMID: 18026103

[ref38] Chuck G. Whipple C. Jackson D. Hake S. (2010). The maize SBP-box transcription factor encoded by tasselsheath4 regulates bract development and the establishment of meristem boundaries. Development 137, 1243–1250. doi: 10.1242/dev.048348, PMID: 20223762

[ref39] Curaba J. Talbot M. Li Z. Helliwell C. (2013). Over-expression of microRNA171 affects phase transitions and floral meristem determinancy in barley. BMC Plant Biol. 13:6. doi: 10.1186/1471-2229-13-6 23294862 PMC3547705

[ref40] D’Ario M. Griffiths-Jones S. Kim M. (2017). Small RNAs: big impact on plant development. Trends Plant Sci. 22, 1056–1068. doi: 10.1016/j.tplants.2017.09.009, PMID: 29032035

[ref41] Dai X. Lu Q. Wang J. Wang L. Xiang F. (2021). MiR160 and its target genes ARF10, ARF16 and ARF17 modulate hypocotyl elongation in a light, BRZ, or PAC-dependent manner in Arabidopsis: MiR160 promotes hypocotyl elongation. Plant Sci. 303:110686. doi: 10.1016/j.plantsci.2020.110686, PMID: 33487334

[ref42] Dai Z. Wang J. Yang X. Lu H. Miao X. (2018). Modulation of plant architecture by the miR156f-OsSPL7-OsGH3.8 pathway in rice. J. Exp. Bot. 69, 5117–5130. doi: 10.1093/jxb/ery273, PMID: 30053063 PMC6184515

[ref43] Dastidar M. G. Scarpa A. Magele I. Ruiz-Duarte P. von Born P. (2019). ARF5/MONOPTEROS directly regulates miR390 expression in the *Arabidopsis thaliana* primary root meristem. Plant Direct 3:e116. doi: 10.1002/pld3.116, PMID: 31245759 PMC6508847

[ref44] Debernardi J. M. Mecchia M. A. Vercruyssen L. Smaczniak C. Kaufmann K. (2014). Post-transcriptional control of GRF transcription factors by microRNA miR396 and GIF co-activator affects leaf size and longevity. Plant J. 79, 413–426. doi: 10.1111/tpj.12567, PMID: 24888433

[ref45] Debernardi J. M. Rodriguez R. E. Mecchia M. A. Palatnik J. F. (2012). Functional specialization of the plant miR396 regulatory network through distinct microRNA-target interactions. PLoS Genet. 8:e1002419. doi: 10.1371/journal.pgen.1002419, PMID: 22242012 PMC3252272

[ref46] Dello I. R. Galinha C. Fletcher A. G. Grigg S. P. Molnar A. (2012). A PHABULOSA/cytokinin feedback loop controls root growth in Arabidopsis. Curr. Biol. 22, 1699–1704. doi: 10.1016/j.cub.2012.07.005, PMID: 22902752

[ref47] Deng P. Muhammad S. Cao M. Wu L. (2018). Biogenesis and regulatory hierarchy of phased small interfering RNAs in plants. Plant Biotechnol. J. 16, 965–975. doi: 10.1111/pbi.12882, PMID: 29327403 PMC5902766

[ref48] Ding Y. Tao Y. Zhu C. (2013). Emerging roles of microRNAs in the mediation of drought stress response in plants. J. Exp. Bot. 64, 3077–3086. doi: 10.1093/jxb/ert164, PMID: 23814278

[ref49] Du F. Gong W. Bosca S. Tucker M. Vaucheret H. (2020). Dose-dependent AGO1-mediated inhibition of the miRNA165/166 pathway modulates stem cell maintenance in arabidopsis shoot apical meristem. Plant Commun. 1:100002. doi: 10.1016/j.xplc.2019.100002, PMID: 33404539 PMC7747967

[ref50] Du Q. Wang H. (2015). The role of HD-ZIP III transcription factors and miR165/166 in vascular development and secondary cell wall formation. Plant Signal. Behav. 10:e1078955. doi: 10.1080/15592324.2015.1078955, PMID: 26340415 PMC4883823

[ref51] Duan C. G. Zhang H. Tang K. Zhu X. Qian W. (2015). Specific but interdependent functions for Arabidopsis AGO4 and AGO6 in RNA-directed DNA methylation. EMBO J. 34, 581–592. doi: 10.15252/embj.201489453, PMID: 25527293 PMC4365029

[ref52] Elbashir S. M. Harborth J. Lendeckel W. Yalcin A. Weber K. (2001). Duplexes of 21-nucleotide RNAs mediate RNA interference in cultured mammalian cells. Nature 411, 494–498. doi: 10.1038/35078107, PMID: 11373684

[ref53] Endo Y. Iwakawa H. O. Tomari Y. (2013). Arabidopsis ARGONAUTE7 selects miR390 through multiple checkpoints during RISC assembly. EMBO Rep. 14, 652–658. doi: 10.1038/embor.2013.73, PMID: 23732541 PMC3701240

[ref54] Fahlgren N. Montgomery T. A. Howell M. D. Allen E. Dvorak S. K. (2006). Regulation of AUXIN RESPONSE FACTOR3 by TAS3 ta-siRNA affects developmental timing and patterning in Arabidopsis. Curr. Biol. 16, 939–944. doi: 10.1016/j.cub.2006.03.065, PMID: 16682356

[ref55] Fan S. Zhang D. Gao C. Wan S. Lei C. (2018). Mediation of flower induction by gibberellin and its inhibitor paclobutrazol: MRNA and miRNA integration comprises complex regulatory cross-talk in apple. Plant Cell Physiol. 59, 2288–2307. doi: 10.1093/pcp/pcy154, PMID: 30137602

[ref56] Fang X. Qi Y. (2016). RNAi in plants: an Argonaute-Centered view. Plant Cell 28, 272–285. doi: 10.1105/tpc.15.00920, PMID: 26869699 PMC4790879

[ref57] Fire A. Xu S. Montgomery M. K. Kostas S. A. Driver S. E. (1998). Potent and specific genetic interference by double-stranded RNA in Caenorhabditis elegans. Nature 391, 806–811. doi: 10.1038/35888, PMID: 9486653

[ref58] Floyd S. K. Bowman J. L. (2004). Gene regulation: ancient microRNA target sequences in plants. Nature 428, 485–486. doi: 10.1038/428485a, PMID: 15057819

[ref59] Fouracre J. P. He J. Chen V. J. Sidoli S. Poethig R. S. (2021). VAL genes regulate vegetative phase change *via* miR156-dependent and independent mechanisms. PLoS Genet. 17:e1009626. doi: 10.1371/journal.pgen.1009626, PMID: 34181637 PMC8270478

[ref60] Gaillochet C. Lohmann J. U. (2015). The never-ending story: From pluripotency to plant developmental plasticity. Development 142, 2237–2249. doi: 10.1242/dev.117614, PMID: 26130755 PMC4510588

[ref61] Guan X. Pang M. Nah G. Shi X. Ye W. (2014). MiR828 and miR858 regulate homoeologous MYB2 gene functions in Arabidopsis trichome and cotton fibre development. Nat. Commun. 5:3050. doi: 10.1038/ncomms4050 24430011

[ref62] Guo C. Jiang Y. Shi M. Wu X. Wu G. (2021). ABI5 acts downstream of miR159 to delay vegetative phase change in Arabidopsis. New Phytol. 231, 339–350. doi: 10.1111/nph.17371, PMID: 33774835

[ref63] Guo H. S. Xie Q. Fei J. F. Chua N. H. (2005). MicroRNA directs mRNA cleavage of the transcription factor NAC1 to downregulate auxin signals for arabidopsis lateral root development. Plant Cell 17, 1376–1386. doi: 10.1105/tpc.105.030841, PMID: 15829603 PMC1091761

[ref64] Guo C. Xu Y. Shi M. Lai Y. Wu X. (2017). Repression of miR156 by miR159 regulates the timing of the juvenile-to-adult transition in arabidopsis. Plant Cell 29, 1293–1304. doi: 10.1105/tpc.16.00975, PMID: 28536099 PMC5502449

[ref65] Gutierrez L. Bussell J. D. Pacurar D. I. Schwambach J. Pacurar M. (2009). Phenotypic plasticity of adventitious rooting in Arabidopsis is controlled by complex regulation of AUXIN RESPONSE FACTOR transcripts and microRNA abundance. Plant Cell 21, 3119–3132. doi: 10.1105/tpc.108.064758 19820192 PMC2782293

[ref66] Ha M. Kim V. N. (2014). Regulation of microRNA biogenesis. Nat. Rev. Mol. Cell Biol. 15, 509–524. doi: 10.1038/nrm3838, PMID: 25027649

[ref67] Hamilton A. J. Baulcombe D. C. (1999). A species of small antisense RNA in posttranscriptional gene silencing in plants. Science 286, 950–952. doi: 10.1126/science.286.5441.950, PMID: 10542148

[ref68] Han J. Fang J. Wang C. Yin Y. Sun X. (2014). Grapevine microRNAs responsive to exogenous gibberellin. BMC Genomics 15:111. doi: 10.1186/1471-2164-15-111 24507455 PMC3937062

[ref69] Han M. H. Goud S. Song L. Fedoroff N. (2004). The Arabidopsis double-stranded RNA-binding protein HYL1 plays a role in microRNA-mediated gene regulation. Proc. Natl. Acad. Sci. U. S. A. 101, 1093–1098. doi: 10.1073/pnas.0307969100 14722360 PMC327156

[ref70] Hay A. Craft J. Tsiantis M. (2004). Plant hormones and homeoboxes: bridging the gap? BioEssays 26, 395–404. doi: 10.1002/bies.20016, PMID: 15057937

[ref71] He J. Xu M. Willmann M. R. McCormick K. Hu T. (2018). Threshold-dependent repression of SPL gene expression by miR156/miR157 controls vegetative phase change in *Arabidopsis thaliana*. PLoS Genet. 14:e1007337. doi: 10.1371/journal.pgen.1007337, PMID: 29672610 PMC5929574

[ref72] Hibara K. Karim M. R. Takada S. Taoka K. Furutani M. (2006). Arabidopsis CUP-SHAPED COTYLEDON3 regulates postembryonic shoot meristem and organ boundary formation. Plant Cell 18, 2946–2957. doi: 10.1105/tpc.106.045716, PMID: 17122068 PMC1693926

[ref73] Huen A. K. Rodriguez-Medina C. Ho A. Atkins C. A. Smith P. (2017). Long-distance movement of phosphate starvation-responsive microRNAs in Arabidopsis. Plant Biol. 19, 643–649. doi: 10.1111/plb.12568, PMID: 28322489

[ref74] Ji L. Liu X. Yan J. Wang W. Yumul R. E. (2011). ARGONAUTE10 and ARGONAUTE1 regulate the termination of floral stem cells through two microRNAs in Arabidopsis. PLoS Genet. 7:e1001358. doi: 10.1371/journal.pgen.1001358, PMID: 21483759 PMC3069122

[ref75] Jia X. Ding N. Fan W. Yan J. Gu Y. (2015). Functional plasticity of miR165/166 in plant development revealed by small tandem target mimic. Plant Sci. 233, 11–21. doi: 10.1016/j.plantsci.2014.12.020, PMID: 25711809

[ref76] Jing X. Rui M. Blake C. (2017). The emergence, evolution, and diversification of the miR390-TAS3-ARF pathway in land plants. Plant Cell 29, 1232–1247. doi: 10.1105/tpc.17.00185, PMID: 28442597 PMC5502456

[ref77] Jodder J. (2020). MiRNA-mediated regulation of auxin signaling pathway during plant development and stress responses. J. Biosci. 45:91. doi: 10.1007/s12038-020-00062-1, PMID: 32713854

[ref78] Jones-Rhoades M. W. Bartel D. P. (2004). Computational identification of plant microRNAs and their targets, including a stress-induced miRNA. Mol. Cell 14, 787–799. doi: 10.1016/j.molcel.2004.05.027, PMID: 15200956

[ref79] Jones-Rhoades M. W. Bartel D. P. Bartel B. (2006). MicroRNAS and their regulatory roles in plants. Annu. Rev. Plant Biol. 57, 19–53. doi: 10.1146/annurev.arplant.57.032905.105218, PMID: 16669754

[ref80] Jung J. H. Park C. M. (2007). MIR166/165 genes exhibit dynamic expression patterns in regulating shoot apical meristem and floral development in Arabidopsis. Planta 225, 1327–1338. doi: 10.1007/s00425-006-0439-1, PMID: 17109148

[ref81] Jung J. H. Seo P. J. Park C. M. (2009). MicroRNA biogenesis and function in higher plants. Plant Biotechnol. Rep. 3, 111–126. doi: 10.1007/s11816-009-0085-8, PMID: 34979922

[ref82] Jung J. H. Seo Y. H. Seo P. J. Reyes J. L. Yun J. (2007). The GIGANTEA-regulated microRNA172 mediates photoperiodic flowering independent of CONSTANS in Arabidopsis. Plant Cell 19, 2736–2748. doi: 10.1105/tpc.107.054528, PMID: 17890372 PMC2048707

[ref83] Kang J. Dengler N. (2002). Cell cycling frequency and expression of the homeobox gene ATHB-8 during leaf vein development in Arabidopsis. Planta 216, 212–219. doi: 10.1007/s00425-002-0847-9, PMID: 12447534

[ref84] Kasschau K. D. Xie Z. Allen E. Llave C. Chapman E. J. (2003). P1/HC-pro, a viral suppressor of RNA silencing, interferes with Arabidopsis development and miRNA unction. Dev. Cell 4, 205–217. doi: 10.1016/S1534-5807(03)00025-X, PMID: 12586064

[ref85] Kidner C. A. Martienssen R. A. (2004). Spatially restricted microRNA directs leaf polarity through ARGONAUTE1. Nature 428, 81–84. doi: 10.1038/nature02366, PMID: 14999284

[ref86] Kim V. N. (2005). Small RNAs: classification, biogenesis, and function. Mol. Cells 19, 1–15. PMID: 15750334

[ref87] Kim J. H. Choi D. Kende H. (2003). The AtGRF family of putative transcription factors is involved in leaf and cotyledon growth in Arabidopsis. Plant J. 36, 94–104. doi: 10.1046/j.1365-313X.2003.01862.x, PMID: 12974814

[ref88] Kim J. Jung J. H. Reyes J. L. Kim Y. S. Kim S. Y. (2005). MicroRNA-directed cleavage of ATHB15 mRNA regulates vascular development in Arabidopsis inflorescence stems. Plant J. 42, 84–94. doi: 10.1111/j.1365-313X.2005.02354.x, PMID: 15773855 PMC1382282

[ref89] Kim J. J. Lee J. H. Kim W. Jung H. S. Huijser P. (2012). The microRNA156-SQUAMOSA PROMOTER BINDING PROTEIN-LIKE3 module regulates ambient temperature-responsive flowering *via* FLOWERING LOCUS T in Arabidopsis. Plant Physiol. 159, 461–478. doi: 10.1104/pp.111.192369, PMID: 22427344 PMC3375978

[ref90] Kim S. Yang J. Y. Xu J. Jang I. C. Prigge M. J. (2008). Two cap-binding proteins CBP20 and CBP80 are involved in processing primary MicroRNAs. Plant Cell Physiol. 49, 1634–1644. doi: 10.1093/pcp/pcn146 18829588 PMC2722234

[ref91] Knauer S. Holt A. L. Rubio-Somoza I. Tucker E. J. Hinze A. (2013). A protodermal miR394 signal defines a region of stem cell competence in the Arabidopsis shoot meristem. Dev. Cell 24, 125–132. doi: 10.1016/j.devcel.2012.12.009, PMID: 23333352

[ref92] Koyama T. Mitsuda N. Seki M. Shinozaki K. Ohme-Takagi M. (2010). TCP transcription factors regulate the activities of ASYMMETRIC LEAVES1 and miR164, as well as the auxin response, during differentiation of LEAVES in Arabidopsis. Plant Cell 22, 3574–3588. doi: 10.1105/tpc.110.075598, PMID: 21119060 PMC3015130

[ref93] Koyama T. Sato F. Ohme-Takagi M. (2017). Roles of miR319 and TCP transcription factors in leaf development. Plant Physiol. 175, 874–885. doi: 10.1104/pp.17.00732, PMID: 28842549 PMC5619901

[ref94] Kumar A. Gautam V. Kumar P. Mukherjee S. Verma S. (2019). Identification and co-evolution pattern of stem cell regulator miR394s and their targets among diverse plant species. BMC Evol. Biol. 19:55. doi: 10.1186/s12862-019-1382-7 30764768 PMC6376759

[ref95] Kurihara Y. Watanabe Y. (2004). Arabidopsis micro-RNA biogenesis through Dicer-like 1 protein functions. Proc. Natl. Acad. Sci. U. S. A. 101, 12753–12758. doi: 10.1073/pnas.0403115101 15314213 PMC515125

[ref96] Kutter C. Schob H. Stadler M. Meins F. J. Si-Ammour A. (2007). MicroRNA-mediated regulation of stomatal development in Arabidopsis. Plant Cell 19, 2417–2429. doi: 10.1105/tpc.107.050377, PMID: 17704216 PMC2002609

[ref97] Laufs P. Peaucelle A. Morin H. Traas J. (2004). MicroRNA regulation of the CUC genes is required for boundary size control in Arabidopsis meristems. Development 131, 4311–4322. doi: 10.1242/dev.01320, PMID: 15294871

[ref98] Lauter N. Kampani A. Carlson S. Goebel M. Moose S. P. (2005). MicroRNA172 down-regulates glossy15 to promote vegetative phase change in maize. Proc. Natl. Acad. Sci. U. S. A. 102, 9412–9417. doi: 10.1073/pnas.0503927102 15958531 PMC1166634

[ref99] Lee R. C. Feinbaum R. L. Ambros V. (1993). The C. Elegans heterochronic gene lin-4 encodes small RNAs with antisense complementarity to lin-14. Cell 75, 843–854. doi: 10.1016/0092-8674(93)90529-Y, PMID: 8252621

[ref100] Lee Y. Kim M. Han J. Yeom K. H. Lee S. (2004). MicroRNA genes are transcribed by RNA polymerase II. EMBO J. 23, 4051–4060. doi: 10.1038/sj.emboj.7600385, PMID: 15372072 PMC524334

[ref101] Leng X. Wang P. Zhu X. Li X. Zheng T. (2017). Ectopic expression of CSD1 and CSD2 targeting genes of miR398 in grapevine is associated with oxidative stress tolerance. Funct. Integr. Genomics 17, 697–710. doi: 10.1007/s10142-017-0565-9, PMID: 28674744

[ref102] Li W. Cui X. Meng Z. Huang X. Xie Q. (2012). Transcriptional regulation of Arabidopsis MIR168a and argonaute1 homeostasis in abscisic acid and abiotic stress responses. Plant Physiol. 158, 1279–1292. doi: 10.1104/pp.111.188789, PMID: 22247272 PMC3291255

[ref103] Li T. Gonzalez N. Inze D. Dubois M. (2020). Emerging connections between small RNAs and Phytohormones. Trends Plant Sci. 25, 912–929. doi: 10.1016/j.tplants.2020.04.004, PMID: 32381482

[ref104] Li W. X. Oono Y. Zhu J. He X. J. Wu J. M. (2008). The Arabidopsis NFYA5 transcription factor is regulated transcriptionally and posttranscriptionally to promote drought resistance. Plant Cell 20, 2238–2251. doi: 10.1105/tpc.108.059444, PMID: 18682547 PMC2553615

[ref105] Li Z. Peng J. Wen X. Guo H. (2013). Ethylene-insensitive3 is a senescence-associated gene that accelerates age-dependent leaf senescence by directly repressing miR164 transcription in Arabidopsis. Plant Cell 25, 3311–3328. doi: 10.1105/tpc.113.113340, PMID: 24064769 PMC3809534

[ref106] Li X. Qian Q. Fu Z. Wang Y. Xiong G. (2003). Control of tillering in rice. Nature 422, 618–621. doi: 10.1038/nature01518, PMID: 12687001

[ref107] Li Y. Tong Y. He X. Zhu Y. Wang W. (2021). The rice miR171b–SCL6-IIs module controls blast resistance, grain yield, and flowering. Crop J. doi: 10.1016/j.cj.2021.05.004, Epub ahead of print

[ref108] Li J. Yang Z. Yu B. Liu J. Chen X. (2005). Methylation protects miRNAs and siRNAs from a 3′-end uridylation activity in Arabidopsis. Curr. Biol. 15, 1501–1507. doi: 10.1016/j.cub.2005.07.029, PMID: 16111943 PMC5127709

[ref109] Lian H. Wang L. Ma N. Zhou C. M. Han L. (2021). Redundant and specific roles of individual MIR172 genes in plant development. PLoS Biol. 19:e3001044. doi: 10.1371/journal.pbio.3001044, PMID: 33529193 PMC7853526

[ref110] Liebsch D. Palatnik J. F. (2020). MicroRNA miR396, GRF transcription factors and GIF co-regulators: A conserved plant growth regulatory module with potential for breeding and biotechnology. Curr. Opin. Plant Biol. 53, 31–42. doi: 10.1016/j.pbi.2019.09.008, PMID: 31726426

[ref801] Lin S. I. Chiang S. F. Lin W. Y. Chen J. W. Tseng C. Y. Wu P. C.. (2008). Regulatory network of microRNA399 and PHO2 by systemic signaling. Plant Physiol. 147, 732–746. doi: 10.1104/pp.108.116269, PMID: 18390805 PMC2409027

[ref111] Liu H. Guo S. Xu Y. Li C. Zhang Z. (2014a). OsmiR396d-regulated OsGRFs function in floral organogenesis in rice through binding to their targets OsJMJ706 and OsCR4. Plant Physiol. 165, 160–174. doi: 10.1104/pp.114.235564, PMID: 24596329 PMC4012577

[ref112] Liu H. Jia S. Shen D. Liu J. Li J. (2012). Four AUXIN RESPONSE FACTOR genes downregulated by microRNA167 are associated with growth and development in Oryza sativa. Funct. Plant Biol. 39, 736–744. doi: 10.1071/FP12106, PMID: 32480824

[ref113] Liu B. Li J. Tsykin A. Liu L. Gaur A. B. (2009a). Exploring complex miRNA-mRNA interactions with Bayesian networks by splitting-averaging strategy. BMC Bioinformatics 10:408. doi: 10.1186/1471-2105-10-408, PMID: 20003267 PMC2797807

[ref114] Liu Z. Li J. Wang L. Li Q. Lu Q. (2016). Repression of callus initiation by the miRNA-directed interaction of auxin-cytokinin in *Arabidopsis thaliana*. Plant J. 87, 391–402. doi: 10.1111/tpj.13211, PMID: 27189514

[ref115] Liu P. P. Montgomery T. A. Fahlgren N. Kasschau K. D. Nonogaki H. (2007). Repression of AUXIN RESPONSE FACTOR10 by microRNA160 is critical for seed germination and post-germination stages. Plant J. 52, 133–146. doi: 10.1111/j.1365-313X.2007.03218.x, PMID: 17672844

[ref116] Liu D. Song Y. Chen Z. Yu D. (2009b). Ectopic expression of miR396 suppresses GRF target gene expression and alters leaf growth in Arabidopsis. Physiol. Plant. 136, 223–236. doi: 10.1111/j.1399-3054.2009.01229.x, PMID: 19453503

[ref117] Liu N. Wu S. Van Houten J. Wang Y. Ding B. (2014b). Down-regulation of AUXIN RESPONSE FACTORS 6 and 8 by microRNA 167 leads to floral development defects and female sterility in tomato. J. Exp. Bot. 65, 2507–2520. doi: 10.1093/jxb/eru141, PMID: 24723401 PMC4036516

[ref118] Liu Q. Yao X. Pi L. Wang H. Cui X. (2009c). The ARGONAUTE10 gene modulates shoot apical meristem maintenance and establishment of leaf polarity by repressing miR165/166 in Arabidopsis. Plant J. 58, 27–40. doi: 10.1111/j.1365-313X.2008.03757.x, PMID: 19054365

[ref119] Llave C. Xie Z. Kasschau K. D. Carrington J. C. (2002). Cleavage of scarecrow-like mRNA targets directed by a class of Arabidopsis miRNA. Science 297, 2053–2056. doi: 10.1126/science.1076311, PMID: 12242443

[ref120] Lopez-Ruiz B. A. Juarez-Gonzalez V. T. Sandoval-Zapotitla E. Dinkova T. D. (2019). Development-related miRNA expression and target regulation during staggered *in vitro* plant regeneration of tuxpeno VS-535 maize cultivar. Int. J. Mol. Sci. 20:2079. doi: 10.3390/ijms20092079, PMID: 31035580 PMC6539278

[ref121] Lu Y. Feng Z. Liu X. Bian L. Xie H. (2018). MiR393 and miR390 synergistically regulate lateral root growth in rice under different conditions. BMC Plant Biol. 18:261. doi: 10.1186/s12870-018-1488-x 30373525 PMC6206659

[ref122] Ma Z. Hu X. Cai W. Huang W. Zhou X. (2014). Arabidopsis miR171-targeted scarecrow-like proteins bind to GT cis-elements and mediate gibberellin-regulated chlorophyll biosynthesis under light conditions. PLoS Genet. 10:e1004519. doi: 10.1371/journal.pgen.1004519, PMID: 25101599 PMC4125095

[ref123] Ma J. Zhao P. Liu S. Yang Q. Guo H. (2020). The control of developmental phase transitions by microRNAs and their targets in seed plants. Int. J. Mol. Sci. 21:1917. doi: 10.3390/ijms21061971, PMID: 32183075 PMC7139601

[ref124] Mallory A. C. Bartel D. P. Bartel B. (2005). MicroRNA-directed regulation of Arabidopsis AUXIN RESPONSE FACTOR17 is essential for proper development and modulates expression of early auxin response genes. Plant Cell 17, 1360–1375. doi: 10.1105/tpc.105.031716, PMID: 15829600 PMC1091760

[ref125] Manuela D. Xu M. (2020). Patterning a leaf by establishing polarities. Front. Plant Sci. 11:568730. doi: 10.3389/fpls.2020.568730, PMID: 33193497 PMC7661387

[ref126] Marin E. Jouannet V. Herz A. Lokerse A. S. Weijers D. (2010). MiR390, Arabidopsis TAS3 tasiRNAs, and their AUXIN RESPONSE FACTOR targets define an autoregulatory network quantitatively regulating lateral root growth. Plant Cell 22, 1104–1117. doi: 10.1105/tpc.109.072553, PMID: 20363771 PMC2879756

[ref127] Martin A. Adam H. Diaz-Mendoza M. Zurczak M. Gonzalez-Schain N. D. (2009). Graft-transmissible induction of potato tuberization by the microRNA miR172. Development 136, 2873–2881. doi: 10.1242/dev.031658, PMID: 19666819

[ref128] Melnyk C. W. Molnar A. Baulcombe D. C. (2011). Intercellular and systemic movement of RNA silencing signals. EMBO J. 30, 3553–3563. doi: 10.1038/emboj.2011.274, PMID: 21878996 PMC3181474

[ref129] Merelo P. Ram H. Pia C. M. Ohno C. Ott F. (2016). Regulation of MIR165/166 by class II and class III homeodomain leucine zipper proteins establishes leaf polarity. Proc. Natl. Acad. Sci. U. S. A. 113, 11973–11978. doi: 10.1073/pnas.1516110113, PMID: 27698117 PMC5081595

[ref130] Millar A. A. Gubler F. (2005). The Arabidopsis GAMYB-like genes, MYB33 and MYB65, are microRNA-regulated genes that redundantly facilitate anther development. Plant Cell 17, 705–721. doi: 10.1105/tpc.104.027920, PMID: 15722475 PMC1069693

[ref131] Millar A. A. Lohe A. Wong G. (2019). Biology and function of miR159 in plants. Plan. Theory 8:255. doi: 10.3390/plants8080255, PMID: 31366066 PMC6724108

[ref132] Montgomery T. A. Howell M. D. Cuperus J. T. Li D. Hansen J. E. (2008). Specificity of ARGONAUTE7-miR390 interaction and dual functionality in TAS3 trans-acting siRNA formation. Cell 133, 128–141. doi: 10.1016/j.cell.2008.02.033, PMID: 18342362

[ref133] Muller C. J. Valdes A. E. Wang G. Ramachandran P. Beste L. (2016). PHABULOSA mediates an auxin signaling loop to regulate vascular patterning in arabidopsis. Plant Physiol. 170, 956–970. doi: 10.1104/pp.15.01204, PMID: 26637548 PMC4734557

[ref134] Muraro D. Mellor N. Pound M. P. Help H. Lucas M. (2014). Integration of hormonal signaling networks and mobile microRNAs is required for vascular patterning in Arabidopsis roots. Proc. Natl. Acad. Sci. U. S. A. 111, 857–862. doi: 10.1073/pnas.1221766111 24381155 PMC3896157

[ref135] Nair S. K. Wang N. Turuspekov Y. Pourkheirandish M. Sinsuwongwat S. (2010). Cleistogamous flowering in barley arises from the suppression of microRNA-guided HvAP2 mRNA cleavage. Proc. Natl. Acad. Sci. U. S. A. 107, 490–495. doi: 10.1073/pnas.0909097107 20018663 PMC2806734

[ref136] Naqvi A. R. Sarwat M. Hasan S. Roychodhury N. (2012). Biogenesis, functions and fate of plant microRNAs. J. Cell. Physiol. 227, 3163–3168. doi: 10.1002/jcp.24052, PMID: 22252306

[ref137] O’Brien J. Hayder H. Zayed Y. Peng C. (2018). Overview of MicroRNA biogenesis, mechanisms of actions, and circulation. Front. Endocrinol. 9:402. doi: 10.3389/fendo.2018.00402 PMC6085463 30123182

[ref138] Ori N. Cohen A. R. Etzioni A. Brand A. Yanai O. (2007). Regulation of LANCEOLATE by miR319 is required for compound-leaf development in tomato. Nat. Genet. 39, 787–791. doi: 10.1038/ng2036, PMID: 17486095

[ref139] Palatnik J. F. Wollmann H. Schommer C. Schwab R. Boisbouvier J. (2007). Sequence and expression differences underlie functional specialization of Arabidopsis microRNAs miR159 and miR319. Dev. Cell 13, 115–125. doi: 10.1016/j.devcel.2007.04.012, PMID: 17609114

[ref140] Pal-Bhadra M. Bhadra U. Birchler J. A. (2002). RNAi related mechanisms affect both transcriptional and posttranscriptional transgene silencing in drosophila. Mol. Cell 9, 315–327. doi: 10.1016/S1097-2765(02)00440-9, PMID: 11864605

[ref802] Pant B. D. Buhtz A. Kehr J. Scheible W. R. (2008). MicroRNA399 is a long-distance signal for the regulation of plant phosphate homeostasis. Plant J. 53, 731–738. doi: 10.1111/j.1365-313X.2007.03363.x, PMID: 17988220 PMC2268993

[ref141] Park M. Y. Wu G. Gonzalez-Sulser A. Vaucheret H. Poethig R. S. (2005). Nuclear processing and export of microRNAs in Arabidopsis. Proc. Natl. Acad. Sci. U. S. A. 102, 3691–3696. doi: 10.1073/pnas.0405570102 15738428 PMC553294

[ref142] Parry G. Calderon-Villalobos L. I. Prigge M. Peret B. Dharmasiri S. (2009). Complex regulation of the TIR1/AFB family of auxin receptors. Proc. Natl. Acad. Sci. U. S. A. 106, 22540–22545. doi: 10.1073/pnas.0911967106 20018756 PMC2799741

[ref143] Piazza P. Jasinski S. Tsiantis M. (2005). Evolution of leaf developmental mechanisms. New Phytol. 167, 693–710. doi: 10.1111/j.1469-8137.2005.01466.x, PMID: 16101907

[ref144] Qi Y. Denli A. M. Hannon G. J. (2005). Biochemical specialization within Arabidopsis RNA silencing pathways. Mol. Cell 19, 421–428. doi: 10.1016/j.molcel.2005.06.014, PMID: 16061187

[ref145] Qi Y. He X. Wang X. J. Kohany O. Jurka J. (2006). Distinct catalytic and non-catalytic roles of ARGONAUTE4 in RNA-directed DNA methylation. Nature 443, 1008–1012. doi: 10.1038/nature05198, PMID: 16998468

[ref146] Qu L. Lin L. B. Xue H. W. (2019). Rice miR394 suppresses LEAF inclination through targeting an F-box gene, LEAF INCLINATION 4. J. Integr. Plant Biol. 61, 406–416. doi: 10.1111/jipb.12713 30144351

[ref147] Ramachandran P. Carlsbecker A. Etchells J. P. (2017). Class III HD-ZIPs govern vascular cell fate: An HD view on patterning and differentiation. J. Exp. Bot. 68, 55–69. doi: 10.1093/jxb/erw370 27794018

[ref148] Raman S. Greb T. Peaucelle A. Blein T. Laufs P. (2008). Interplay of miR164, CUP-SHAPED COTYLEDON genes and LATERAL SUPPRESSOR controls axillary meristem formation in *Arabidopsis thaliana*. Plant J. 55, 65–76. doi: 10.1111/j.1365-313X.2008.03483.x, PMID: 18346190

[ref149] Rodriguez R. E. Mecchia M. A. Debernardi J. M. Schommer C. Weigel D. (2010). Control of cell proliferation in *Arabidopsis thaliana* by microRNA miR396. Development 137, 103–112. doi: 10.1242/dev.043067, PMID: 20023165 PMC2796936

[ref150] Ru P. Xu L. Ma H. Huang H. (2006). Plant fertility defects induced by the enhanced expression of microRNA167. Cell Res. 16, 457–465. doi: 10.1038/sj.cr.7310057, PMID: 16699541

[ref151] Rubio-Somoza I. Weigel D. (2013). Coordination of flower maturation by a regulatory circuit of three microRNAs. PLoS Genet. 9:e1003374. doi: 10.1371/journal.pgen.1003374, PMID: 23555288 PMC3610633

[ref152] Salvi S. Sponza G. Morgante M. Tomes D. Niu X. (2007). Conserved noncoding genomic sequences associated with a flowering-time quantitative trait locus in maize. Proc. Natl. Acad. Sci. U. S. A. 104, 11376–11381. doi: 10.1073/pnas.0704145104 17595297 PMC2040906

[ref153] Schommer C. Debernardi J. M. Bresso E. G. Rodriguez R. E. Palatnik J. F. (2014). Repression of cell proliferation by miR319-regulated TCP4. Mol. Plant 7, 1533–1544. doi: 10.1093/mp/ssu084, PMID: 25053833

[ref154] Schoof H. Lenhard M. Haecker A. Mayer K. F. Jurgens G. (2000). The stem cell population of Arabidopsis shoot meristems in maintained by a regulatory loop between the CLAVATA and WUSCHEL genes. Cell 100, 635–644. doi: 10.1016/S0092-8674(00)80700-X, PMID: 10761929

[ref155] Schwab R. Palatnik J. F. Riester M. Schommer C. Schmid M. (2005). Specific effects of microRNAs on the plant transcriptome. Dev. Cell 8, 517–527. doi: 10.1016/j.devcel.2005.01.018, PMID: 15809034

[ref156] Scofield S. Dewitte W. Murray J. A. (2014). STM sustains stem cell function in the Arabidopsis shoot apical meristem and controls KNOX gene expression independently of the transcriptional repressor AS1. Plant Signal. Behav. 9:e28934. doi: 10.4161/psb.28934, PMID: 24776954 PMC4091562

[ref157] Sijen T. Vijn I. Rebocho A. van Blokland R. Roelofs D. (2001). Transcriptional and posttranscriptional gene silencing are mechanistically related. Curr. Biol. 11, 436–440. doi: 10.1016/S0960-9822(01)00116-6, PMID: 11301254

[ref158] Singh R. K. Gase K. Baldwin I. T. Pandey S. P. (2015). Molecular evolution and diversification of the Argonaute family of proteins in plants. BMC Plant Biol. 15:23. doi: 10.1186/s12870-014-0364-6 25626325 PMC4318128

[ref159] Skopelitis D. S. Benkovics A. H. Husbands A. Y. Timmermans M. (2017). Boundary formation through a direct threshold-based readout of mobile small RNA gradients. Dev. Cell 43, 265–273. doi: 10.1016/j.devcel.2017.10.003, PMID: 29107557

[ref160] Skopelitis D. S. Hill K. Klesen S. Marco C. F. von Born P. (2018). Gating of miRNA movement at defined cell-cell interfaces governs their impact as positional signals. Nat. Commun. 9:3107. doi: 10.1038/s41467-018-05571-0 30082703 PMC6079027

[ref161] Somssich M. Je B. I. Simon R. Jackson D. (2016). CLAVATA-WUSCHEL signaling in the shoot meristem. Development 143, 3238–3248. doi: 10.1242/dev.133645, PMID: 27624829

[ref162] Song J. B. Huang S. Q. Dalmay T. Yang Z. M. (2012). Regulation of LEAF morphology by microRNA394 and its target LEAF CURLING RESPONSIVENESS. Plant Cell Physiol. 53, 1283–1294. doi: 10.1093/pcp/pcs080, PMID: 22619471

[ref163] Song X. Li Y. Cao X. Qi Y. (2019). MicroRNAs and their regulatory roles in plant-environment interactions. Annu. Rev. Plant Biol. 70, 489–525. doi: 10.1146/annurev-arplant-050718-100334, PMID: 30848930

[ref164] Sorin C. Declerck M. Christ A. Blein T. Ma L. (2014). A miR169 isoform regulates specific NF-YA targets and root architecture in Arabidopsis. New Phytol. 202, 1197–1211. doi: 10.1111/nph.12735, PMID: 24533947

[ref165] Stricklin S. L. Griffiths-Jones S. Eddy S. R. (2005). C. Elegans noncoding RNA genes. WormBook 25, 1–7. doi: 10.1895/wormbook.1.1.1, PMID: 18023116 PMC4781554

[ref166] Su Z. Wang N. Hou Z. Li B. Li D. (2020). Regulation of female germline specification *via* small RNA mobility in arabidopsis. Plant Cell 32, 2842–2854. doi: 10.1105/tpc.20.00126, PMID: 32703817 PMC7474286

[ref167] Sun Z. Li M. Zhou Y. Guo T. Liu Y. (2018). Coordinated regulation of Arabidopsis microRNA biogenesis and red light signaling through Dicer-like 1 and phytochrome-interacting factor 4. PLoS Genet. 14:e1007247. doi: 10.1371/journal.pgen.1007247, PMID: 29522510 PMC5862502

[ref168] Sun X. Wang C. Xiang N. Li X. Yang S. (2017). Activation of secondary cell wall biosynthesis by miR319-targeted TCP4 transcription factor. Plant Biotechnol. J. 15, 1284–1294. doi: 10.1111/pbi.12715, PMID: 28233945 PMC5595714

[ref169] Sunkar R. Zhu J. K. (2004). Novel and stress-regulated microRNAs and other small RNAs from Arabidopsis. Plant Cell 16, 2001–2019. doi: 10.1105/tpc.104.022830, PMID: 15258262 PMC519194

[ref170] Szczygiel-Sommer A. Gaj M. D. (2019). The miR396-GRF regulatory module controls the embryogenic response in arabidopsis *via* an auxin-related pathway. Int. J. Mol. Sci. 20:5221. doi: 10.3390/ijms20205221, PMID: 31640280 PMC6829408

[ref171] Tamiru M. Hardcastle T. J. Lewsey M. G. (2018). Regulation of genome-wide DNA methylation by mobile small RNAs. New Phytol. 217, 540–546. doi: 10.1111/nph.14874, PMID: 29105762

[ref172] Tang G. Yan J. Gu Y. Qiao M. Fan R. (2012). Construction of short tandem target mimic (STTM) to block the functions of plant and animal microRNAs. Methods 58, 118–125. doi: 10.1016/j.ymeth.2012.10.006, PMID: 23098881 PMC3631596

[ref173] Tatematsu K. Toyokura K. Okada K. (2015). Requirement of MIR165A primary transcript sequence for its activity pattern in Arabidopsis leaf primordia. Plant Signal. Behav. 10:e1055432. doi: 10.1080/15592324.2015.1055432, PMID: 26177565 PMC4623492

[ref174] Tirumalai V. Swetha C. Nair A. Pandit A. Shivaprasad P. V. (2019). MiR828 and miR858 regulate VvMYB114 to promote anthocyanin and flavonol accumulation in grapes. J. Exp. Bot. 70, 4775–4792. doi: 10.1093/jxb/erz264, PMID: 31145783 PMC6760283

[ref175] Tsikou D. Yan Z. Holt D. B. Abel N. B. Reid D. E. (2018). Systemic control of legume susceptibility to rhizobial infection by a mobile microRNA. Science 362, 233–236. doi: 10.1126/science.aat6907, PMID: 30166437

[ref176] Tsuji H. Aya K. Ueguchi-Tanaka M. Shimada Y. Nakazono M. (2006). GAMYB controls different sets of genes and is differentially regulated by microRNA in aleurone cells and anthers. Plant J. 47, 427–444. doi: 10.1111/j.1365-313X.2006.02795.x, PMID: 16792694

[ref177] Vaten A. Dettmer J. Wu S. Stierhof Y. D. Miyashima S. (2011). Callose biosynthesis regulates symplastic trafficking during root development. Dev. Cell 21, 1144–1155. doi: 10.1016/j.devcel.2011.10.006, PMID: 22172675

[ref178] Vaucheret H. (2008). Plant ARGONAUTES. Trends Plant Sci. 13, 350–358. doi: 10.1016/j.tplants.2008.04.007, PMID: 18508405

[ref179] Veit B. (2009). Hormone mediated regulation of the shoot apical meristem. Plant Mol. Biol. 69, 397–408. doi: 10.1007/s11103-008-9396-3, PMID: 18797999

[ref180] Voinnet O. (2009). Origin, biogenesis, and activity of plant microRNAs. Cell 136, 669–687. doi: 10.1016/j.cell.2009.01.046, PMID: 19239888

[ref181] Waheed S. Zeng L. (2020). The critical role of miRNAs in regulation of flowering time and flower development. Genes 11:319. doi: 10.3390/genes11030319, PMID: 32192095 PMC7140873

[ref182] Wang J. Bao J. Zhou B. Li M. Li X. (2021b). The Osa-miR164 target OsCUC1 functions redundantly with OsCUC3 in controlling rice meristem/organ boundary specification. New Phytol. 229, 1566–1581. doi: 10.1111/nph.16939, PMID: 32964416 PMC7821251

[ref183] Wang L. Gu X. Xu D. Wang W. Wang H. (2011). MiR396-targeted AtGRF transcription factors are required for coordination of cell division and differentiation during leaf development in Arabidopsis. J. Exp. Bot. 62, 761–773. doi: 10.1093/jxb/erq307, PMID: 21036927 PMC3003814

[ref184] Wang J. J. Guo H. S. (2015). Cleavage of INDOLE-3-ACETIC ACID INDUCIBLE28 mRNA by microRNA847 upregulates auxin signaling to modulate cell proliferation and lateral organ growth in Arabidopsis. Plant Cell 27, 574–590. doi: 10.1105/tpc.15.00101, PMID: 25794935 PMC4558675

[ref185] Wang H. Li Y. Chern M. Zhu Y. Zhang L. L. (2021a). Suppression of rice miR168 improves yield, flowering time and immunity. Nat. Plants 7, 129–136. doi: 10.1038/s41477-021-00852-x, PMID: 33594262

[ref186] Wang L. Liu Z. Qiao M. Xiang F. (2018). MiR393 inhibits *in vitro* shoot regeneration in *Arabidopsis thaliana via* repressing TIR1. Plant Sci. 266, 1–8. doi: 10.1016/j.plantsci.2017.10.009, PMID: 29241559

[ref187] Wang J. W. Schwab R. Czech B. Mica E. Weigel D. (2008). Dual effects of miR156-targeted SPL genes and CYP78A5/KLUH on plastochron length and organ size in *Arabidopsis thaliana*. Plant Cell 20, 1231–1243. doi: 10.1105/tpc.108.058180, PMID: 18492871 PMC2438454

[ref188] Wang H. Wang H. (2015). The miR156/SPL module, a regulatory hub and versatile toolbox, gears up crops for enhanced agronomic traits. Mol. Plant 8, 677–688. doi: 10.1016/j.molp.2015.01.008, PMID: 25617719

[ref189] Wang J. W. Wang L. J. Mao Y. B. Cai W. J. Xue H. W. (2005). Control of root cap formation by MicroRNA-targeted auxin response factors in Arabidopsis. Plant Cell 17, 2204–2216. doi: 10.1105/tpc.105.033076, PMID: 16006581 PMC1182483

[ref190] Wang R. Yang X. Guo S. Wang Z. Zhang Z. (2021c). MiR319-targeted OsTCP21 and OsGAmyb regulate tillering and grain yield in rice. J. Integr. Plant Biol. 63, 1260–1272. doi: 10.1111/jipb.13097, PMID: 33838011

[ref191] Wang T. Zheng Y. Tang Q. Zhong S. Su W. (2021d). Brassinosteroids inhibit miRNA-mediated translational repression by decreasing AGO1 on the endoplasmic reticulum. J. Integr. Plant Biol. 63, 1475–1490. doi: 10.1111/jipb.13139, PMID: 34020507

[ref192] Werner S. Bartrina I. Schmulling T. (2021). Cytokinin regulates vegetative phase change in *Arabidopsis thaliana* through the miR172/TOE1-TOE2 module. Nat. Commun. 12:5816. doi: 10.1038/s41467-021-26088-z 34611150 PMC8492644

[ref193] Wightman B. Ha I. Ruvkun G. (1993). Posttranscriptional regulation of the heterochronic gene lin-14 by lin-4 mediates temporal pattern formation in C Elegans. Cell 75, 855–862. doi: 10.1016/0092-8674(93)90530-4, PMID: 8252622

[ref194] Williams L. Grigg S. P. Xie M. Christensen S. Fletcher J. C. (2005). Regulation of Arabidopsis shoot apical meristem and lateral organ formation by microRNA miR166g and its AtHD-ZIP target genes. Development 132, 3657–3668. doi: 10.1242/dev.01942, PMID: 16033795

[ref195] Windels D. Vazquez F. (2011). MiR393: integrator of environmental cues in auxin signaling? Plant Signal. Behav. 6, 1672–1675. doi: 10.4161/psb.6.11.17900 22067993 PMC3329333

[ref196] Wollmann H. Mica E. Todesco M. Long J. A. Weigel D. (2010). On reconciling the interactions between APETALA2, miR172 and AGAMOUS with the ABC model of flower development. Development 137, 3633–3642. doi: 10.1242/dev.036673, PMID: 20876650 PMC2964095

[ref197] Wu G. Park M. Y. Conway S. R. Wang J. W. Weigel D. (2009). The sequential action of miR156 and miR172 regulates developmental timing in Arabidopsis. Cell 138, 750–759. doi: 10.1016/j.cell.2009.06.031, PMID: 19703400 PMC2732587

[ref198] Wu G. Poethig R. S. (2006). Temporal regulation of shoot development in *Arabidopsis thaliana* by miR156 and its target SPL3. Development 133, 3539–3547. doi: 10.1242/dev.02521, PMID: 16914499 PMC1610107

[ref199] Wu M. F. Tian Q. Reed J. W. (2006). Arabidopsis microRNA167 controls patterns of ARF6 and ARF8 expression, and regulates both female and male reproduction. Development 133, 4211–4218. doi: 10.1242/dev.02602, PMID: 17021043

[ref200] Xia R. Xu J. Meyers B. C. (2017). The emergence, evolution, and diversification of the miR390-TAS3-ARF pathway in land plants. Plant Cell 29, 1232–1247. doi: 10.1105/tpc.17.00185, PMID: 28442597 PMC5502456

[ref201] Xie K. Wu C. Xiong L. (2006). Genomic organization, differential expression, and interaction of SQUAMOSA promoter-binding-like transcription factors and microRNA156 in rice. Plant Physiol. 142, 280–293. doi: 10.1104/pp.106.084475, PMID: 16861571 PMC1557610

[ref202] Xing L. Zhu M. Luan M. Zhang M. Jin L. (2021). MiR169q and NUCLEAR FACTOR YA8 enhance salt tolerance by activating PEROXIDASE1 expression in response to ROS. Plant Physiol. 188, 608–623. doi: 10.1093/plphys/kiab498, PMID: 34718783 PMC8774724

[ref203] Xu M. Hu T. Smith M. R. Poethig R. S. (2016a). Epigenetic regulation of vegetative phase change in arabidopsis. Plant Cell 28, 28–41. doi: 10.1105/tpc.15.00854, PMID: 26704382 PMC4746683

[ref204] Xu M. Hu T. Zhao J. Park M. Y. Earley K. W. (2016b). Developmental functions of miR156-regulated SQUAMOSA PROMOTER BINDING PROTEIN-LIKE (SPL) genes in *Arabidopsis thaliana*. PLoS Genet. 12:e1006263. doi: 10.1371/journal.pgen.1006263, PMID: 27541584 PMC4991793

[ref205] Xu M. Y. Zhang L. Li W. W. Hu X. L. Wang M. B. (2014). Stress-induced early flowering is mediated by miR169 in *Arabidopsis thaliana*. J. Exp. Bot. 65, 89–101. doi: 10.1093/jxb/ert353, PMID: 24336445

[ref206] Yadav A. Kumar S. Verma R. Lata C. Sanyal I. (2021). MicroRNA 166: An evolutionarily conserved stress biomarker in land plants targeting HD-ZIP family. Physiol. Mol. Biol. Plants 27, 2471–2485. doi: 10.1007/s12298-021-01096-x, PMID: 34924705 PMC8639965

[ref207] Yan J. Wang P. Wang B. Hsu C. C. Tang K. (2017). The SnRK2 kinases modulate miRNA accumulation in Arabidopsis. PLoS Genet. 13:e1006753. doi: 10.1371/journal.pgen.1006753, PMID: 28419088 PMC5413060

[ref208] Yan J. Zhao C. Zhou J. Yang Y. Wang P. (2016). The miR165/166 mediated regulatory module plays critical roles in ABA homeostasis and response in *Arabidopsis thaliana*. PLoS Genet. 12:e1006416. doi: 10.1371/journal.pgen.1006416, PMID: 27812104 PMC5094776

[ref209] Yanai O. Shani E. Russ D. Ori N. (2011). Gibberellin partly mediates LANCEOLATE activity in tomato. Plant J. 68, 571–582. doi: 10.1111/j.1365-313X.2011.04716.x, PMID: 21771122

[ref210] Yang T. Wang Y. Teotia S. Wang Z. Shi C. (2019). The interaction between miR160 and miR165/166 in the control of leaf development and drought tolerance in Arabidopsis. Sci. Rep. 9:2832. doi: 10.1038/s41598-019-39397-7 30808969 PMC6391385

[ref211] Yao X. Chen J. Zhou J. Yu H. Ge C. (2019). An essential role for miRNA167 in maternal control of embryonic and seed development. Plant Physiol. 180, 453–464. doi: 10.1104/pp.19.00127, PMID: 30867333 PMC6501067

[ref212] Yokotani N. Nakano R. Imanishi S. Nagata M. Inaba A. (2009). Ripening-associated ethylene biosynthesis in tomato fruit is autocatalytically and developmentally regulated. J. Exp. Bot. 60, 3433–3442. doi: 10.1093/jxb/erp185, PMID: 19605457 PMC2724697

[ref213] Yoon E. K. Yang J. H. Lim J. Kim S. H. Kim S. K. (2010). Auxin regulation of the microRNA390-dependent transacting small interfering RNA pathway in Arabidopsis lateral root development. Nucleic Acids Res. 38, 1382–1391. doi: 10.1093/nar/gkp1128, PMID: 19969544 PMC2831332

[ref214] Yu S. Galvao V. C. Zhang Y. C. Horrer D. Zhang T. Q. (2012). Gibberellin regulates the Arabidopsis floral transition through miR156-targeted SQUAMOSA promoter binding-like transcription factors. Plant Cell 24, 3320–3332. doi: 10.1105/tpc.112.101014, PMID: 22942378 PMC3462634

[ref215] Yu Y. Jia T. Chen X. (2017). The ‘how’ and ‘where’ of plant microRNAs. New Phytol. 216, 1002–1017. doi: 10.1111/nph.14834, PMID: 29048752 PMC6040672

[ref216] Yu S. Wang J. W. (2020). The crosstalk between MicroRNAs and gibberellin Signaling in plants. Plant Cell Physiol. 61, 1880–1890. doi: 10.1093/pcp/pcaa079, PMID: 32845336

[ref217] Yu L. Yu X. Shen R. He Y. (2005). HYL1 gene maintains venation and polarity of leaves. Planta 221, 231–242. doi: 10.1007/s00425-004-1439-7, PMID: 15580355

[ref218] Zhai J. Jeong D. H. De Paoli E. Park S. Rosen B. D. (2011). MicroRNAs as master regulators of the plant NB-LRR defense gene family *via* the production of phased, trans-acting siRNAs. Genes Dev. 25, 2540–2553. doi: 10.1101/gad.177527.111, PMID: 22156213 PMC3243063

[ref219] Zhang X. Henderson I. R. Lu C. Green P. J. Jacobsen S. E. (2007). Role of RNA polymerase IV in plant small RNA metabolism. Proc. Natl. Acad. Sci. U. S. A. 104, 4536–4541. doi: 10.1073/pnas.0611456104 17360559 PMC1810338

[ref220] Zhang T. Q. Lian H. Tang H. Dolezal K. Zhou C. M. (2015). An intrinsic microRNA timer regulates progressive decline in shoot regenerative capacity in plants. Plant Cell 27, 349–360. doi: 10.1105/tpc.114.135186, PMID: 25649435 PMC4456919

[ref221] Zhang B. Pan X. Stellwag E. J. (2008). Identification of soybean microRNAs and their targets. Planta 229, 161–182. doi: 10.1007/s00425-008-0818-x, PMID: 18815805

[ref222] Zhang F. Wang L. Lim J. Y. Kim T. Pyo Y. (2016). Phosphorylation of CBP20 links MicroRNA to root growth in the ethylene response. PLoS Genet. 12:e1006437. doi: 10.1371/journal.pgen.1006437, PMID: 27870849 PMC5147770

[ref223] Zhang Y. C. Yu Y. Wang C. Y. Li Z. Y. Liu Q. (2013). Overexpression of microRNA OsmiR397 improves rice yield by increasing grain size and promoting panicle branching. Nat. Biotechnol. 31, 848–852. doi: 10.1038/nbt.2646, PMID: 23873084

[ref224] Zhang H. Zhang L. Han J. Qian Z. Zhou B. (2019). The nuclear localization signal is required for the function of squamosa promoter binding protein-like gene 9 to promote vegetative phase change in Arabidopsis. Plant Mol. Biol. 100, 571–578. doi: 10.1007/s11103-019-00863-5, PMID: 30953277

[ref225] Zhang J. Zhou Z. Bai J. Tao X. Wang L. (2020). Disruption of MIR396e and MIR396f improves rice yield under nitrogen-deficient conditions. Natl. Sci. Rev. 7, 102–112. doi: 10.1093/nsr/nwz142 34692021 PMC8288854

[ref226] Zhao B. Ge L. Liang R. Li W. Ruan K. (2009). Members of miR-169 family are induced by high salinity and transiently inhibit the NF-YA transcription factor. BMC Mol. Biol. 10:29. doi: 10.1186/1471-2199-10-29 19351418 PMC2670843

[ref227] Zhao Y. Lin S. Qiu Z. Cao D. Wen J. (2015). MicroRNA857 is involved in the regulation of secondary growth of vascular tissues in arabidopsis. Plant Physiol. 169, 2539–2552. doi: 10.1104/pp.15.01011, PMID: 26511915 PMC4677895

[ref228] Zhao Y. F. Peng T. Sun H. Z. Teotia S. Wen H. L. (2019). MiR1432-OsACOT (acyl-CoA thioesterase) module determines grain yield *via* enhancing grain filling rate in rice. Plant Biotechnol. J. 17, 712–723. doi: 10.1111/pbi.13009, PMID: 30183128 PMC6419572

[ref229] Zheng L. Zhang X. Zhang H. Gu Y. Huang X. (2019). The miR164-dependent regulatory pathway in developing maize seed. Mol. Gen. Genomics. 294, 501–517. doi: 10.1007/s00438-018-1524-4, PMID: 30607602

[ref230] Zhong R. Ye Z. H. (1999). IFL1, a gene regulating interfascicular fiber differentiation in Arabidopsis, encodes a homeodomain-leucine zipper protein. Plant Cell 11, 2139–2152. doi: 10.1105/tpc.11.11.2139, PMID: 10559440 PMC144121

[ref231] Zhong R. Ye Z. H. (2004). Amphivasal vascular bundle 1, a gain-of-function mutation of the IFL1/REV gene, is associated with alterations in the polarity of leaves, stems and carpels. Plant Cell Physiol. 45, 369–385. doi: 10.1093/pcp/pch051, PMID: 15111711

[ref232] Zhou G. K. Kubo M. Zhong R. Demura T. Ye Z. H. (2007). Overexpression of miR165 affects apical meristem formation, organ polarity establishment and vascular development in Arabidopsis. Plant Cell Physiol. 48, 391–404. doi: 10.1093/pcp/pcm008, PMID: 17237362

[ref233] Zhou X. Wang G. Sutoh K. Zhu J. K. Zhang W. (2008). Identification of cold-inducible microRNAs in plants by transcriptome analysis. Biochim. Biophys. Acta 1779, 780–788. doi: 10.1016/j.bbagrm.2008.04.005 18471443

[ref234] Zhu Q. H. Helliwell C. A. (2011). Regulation of flowering time and floral patterning by miR172. J. Exp. Bot. 62, 487–495. doi: 10.1093/jxb/erq295, PMID: 20952628

[ref235] Zhu H. Hu F. Wang R. Zhou X. Sze S. H. (2011). Arabidopsis Argonaute10 specifically sequesters miR166/165 to regulate shoot apical meristem development. Cell 145, 242–256. doi: 10.1016/j.cell.2011.03.024 21496644 PMC4124879

[ref236] Zhu Q. H. Upadhyaya N. M. Gubler F. Helliwell C. A. (2009). Over-expression of miR172 causes loss of spikelet determinacy and floral organ abnormalities in rice (Oryza sativa). BMC Plant Biol. 9:149. doi: 10.1186/1471-2229-9-149 20017947 PMC2803185

